# From genome to gene module: decoding the BsAlfin2-BsTT2 regulatory network controlling low temperature-induced anthocyanin biosynthesis in *Begonia semperflorens*

**DOI:** 10.1093/hr/uhag319

**Published:** 2026-08-06

**Authors:** Lingyu Song, Zhirou Liu, Yixue Zhang, Puyu Ren, Fei Li, Qirui Wang, Yonghua Li, Fude Shang, Kaiming Zhang

**Affiliations:** College of Landscape Architecture, Henan Agricultural University, Zhengzhou, Henan 450002, China; College of Landscape Architecture, Henan Agricultural University, Zhengzhou, Henan 450002, China; College of Landscape Architecture, Henan Agricultural University, Zhengzhou, Henan 450002, China; College of Landscape Architecture, Henan Agricultural University, Zhengzhou, Henan 450002, China; College of Landscape Architecture, Henan Agricultural University, Zhengzhou, Henan 450002, China; College of Landscape Architecture, Henan Agricultural University, Zhengzhou, Henan 450002, China; College of Landscape Architecture, Henan Agricultural University, Zhengzhou, Henan 450002, China; College of Life Sciences, Henan Agricultural University, Zhengzhou, Henan 450046, China; College of Landscape Architecture, Henan Agricultural University, Zhengzhou, Henan 450002, China

## Abstract

*Begonia semperflorens* is an important ornamental plant worldwide, but its practical application is severely limited by low temperature sensitivity. However, the molecular regulatory mechanisms underlying low temperature-induced anthocyanin biosynthesis remain unclear. In this study, we assembled a high-quality chromosome-level *B. semperflorens* genome. Through genomic and MYB gene family analysis, we identified a key transcription factor *BsTT2*, which directly binds to the *BsDFR* promoter and enhances its activity, thereby driving anthocyanin accumulation in *B. semperflorens*. Furthermore, using BsTT2 as bait, we identified its interacting protein BsAlfin2. Under low temperature-induced reactive oxygen species (ROS) signaling, BsAlfin2 undergoes nuclear translocation and forms a complex with BsTT2, synergistically enhancing the activation of the *BsDFR* promoter and significantly improving anthocyanin production efficiency. Based on these results, we propose a previously uncharacterized BsAlfin2/BsTT2-BsDFR regulatory module, which reveals a molecular links low-temperature ROS signaling to anthocyanin biosynthesis in *B. semperflorens*. In summary, this study not only provides chromosome-level genomic resources for *B. semperflorens* research but also elucidates a key molecular module in low temperature-induced anthocyanin biosynthesis regulatory pathway, laying a theoretical and data foundation for future studies on leaf color improvement and stress resistance breeding in *Begonia*.

## Introduction

*Begonias* are widely appreciated horticultural plants, prized for their diverse morphology and vibrant leaf colors. They are extensively utilized in various applications, including ornamental bedding and indoor foliage [1]. *Begonia semperflorens* is a perennial evergreen herbaceous plant in the family Begoniaceae [2]. With a breeding history spanning nearly a century, it has given rise to a wide range of cultivars renowned for their variations in flower color, flower form, and foliage characteristics [3]. Notably, *B. semperflorens* is considered one of the most low-temperature-tolerant cultivars among begonias [2]. Distinctive leaf variegation represents a key ornamental trait in *B. semperflorens* and is highly valued in commercial markets [4, 5]. Distinctive leaf variegation may result from multiple mechanisms, such as differences in anthocyanin accumulation, chlorophyll deficiency, spatial variation in carotenoids, or other pigment distributional variations [6]. Notably, anthocyanin accumulation stands out as a particularly significant and frequent driver of this trait [7]. Therefore, investigating the regulatory mechanism of anthocyanin biosynthesis in *B. semperflorens* is essential for developing new cultivars with enhanced ornamental appeal.

Anthocyanins, vital secondary metabolites, not only enhance physiological functions such as stress resistance but also contribute to ornamental phenotypes [8]. MYB transcription factors represent one of the largest transcription factor (TF) families in plants, with the R2R3-MYB subfamily playing a particularly important role in the regulation of anthocyanin synthesis [9–11]. These R2R3-MYBs often form the MYB-bHLH-WD40 (MBW) complex together with bHLH and WD40 proteins [12]. The MBW complex precisely modulates the spatiotemporal accumulation of anthocyanins by directly activating or repressing structural genes (*CHS*, *DFR*, and *ANS*) through binding to their promoter regions [13]. Beyond the classical MBW complex model, MYB transcription factors can regulate anthocyanin biosynthesis through various alternative mechanisms. For instance, Luo *et al*. [11] reported that FeR2R3-MYB interacts with FeLAR to promote anthocyanin biosynthesis and enhance drought tolerance in common buckwheat. Du *et al*. [9] demonstrated that two tandemly arranged transcription factors, StMYB11400 and StMYB11410, coordinately regulate anthocyanin accumulation in potato tubers. In addition, a critical gap exists in our understanding of how R2R3-MYB transcription factors regulate the unique anthocyanin pigmentation in *B. semperflorens*.

Numerous studies have indicated that reactive oxygen species (ROS) can induce MYB transcription factors to regulate anthocyanin biosynthesis in plants [14, 15]. For instance, Zhang *et al*. [14] revealed that ROS regulated anthocyanin biosynthesis in pear under high-light stress through the PP2A-HB40-MYB123-like module. Similarly, during lychee fruit maturation, ROS have been shown to upregulate the expression of *LcMYB1*, thereby promoting anthocyanin accumulation [15]. In *B. semperflorens*, our previous study demonstrated that exogenous application of a ROS scavenger at low temperatures suppressed anthocyanin accumulation, in contrast to the promotive effect observed following treatment with hydrogen peroxide (H_2_O_2_) or nicotinamide adenine dinucleotide phosphate on the leaf margins [16]. However, how ROS mediate anthocyanin biosynthesis in *B. semperflorens* at the molecular level has not been elucidated.

The *Alfin* family genes has been demonstrated to play a functional role in mediating plant responses to various abiotic stresses, specifically salt and drought [17, 18]. Recent research has demonstrated that *GmAlfin09* reduces ROS levels by promoting the expression of the ROS-clearing protein *GmPRDX6*, thereby enhancing soybean stress resistance [19]. Further, previous studies on flavonoid accumulation have shown that the *Alfin* gene family is associated with flavonoid accumulation in *Citrus reticulata* peel [20]. Although recent studies have implicated the *Alfin* gene family in ROS signaling and stress tolerance, and ROS have been shown to promote anthocyanin biosynthesis in plants, their regulatory mechanisms in *B. semperflorens* remain unclear. Specifically, how they mediate anthocyanin biosynthesis and low-temperature response awaits further elucidation. In this study, we present the first chromosome-level genome assembly of *B. semperflorens*. Subsequently, *MYB* gene family members were systematically identified from the genome. Functional analysis established *BsTT2* as a key transcriptional regulator of anthocyanin biosynthesis in *B. semperflorens*. Furthermore, we elucidated the molecular mechanism by which ROS mediate anthocyanin biosynthesis under low-temperature stress through the BsAlfin2/BsTT2-BsDFR regulatory module. These findings provide crucial theoretical foundations and genetic resources for advancing genetic breeding programs in *B. semperflorens* and for deciphering its anthocyanin biosynthesis regulatory network.

## Results

### Low temperature induces anthocyanin biosynthesis in *B. semperflorens*

We observed distinct phenotypic changes in *B. semperflorens* under low-temperature conditions. On the third day of low-temperature treatment, the leaves exhibited obvious reddening, which persisted throughout the treatment period (Fig. S1). Consistent with this visual phenotype, quantitative analysis revealed a significant increase in anthocyanin content beginning on the third day, with levels continuing to rise as the treatment progressed (Fig. S2).

### Genome assembly and annotation of *B. semperflorens*

Based on the *k*-mer 23 counts, we estimated that the total genome size of *B. semperflorens* was ~240.60 Mb (Fig. S3). *B. semperflorens* is characterized as an ultra-high-heterozygosity and highly repetitive genome. Sequencing on the DNBseq platform yielded 27.87 Gb Hi-C clean data (Table S1). Total 259.68 Mb sequences were pinned onto 17 chromosomes, accounting for 96.04% of the initial assembly (Fig. 1A, Table S2). The chromosome-scale genome assembly comprises 29 scaffolds, with an N50 of 14.43 Mb and the longest scaffold being 24.87 Mb (Table S3). The assembly achieved 98% completeness according to the Benchmarking Universal Single-Copy Orthologs (BUSCO) assessment (Table S4).

**Figure 1:**
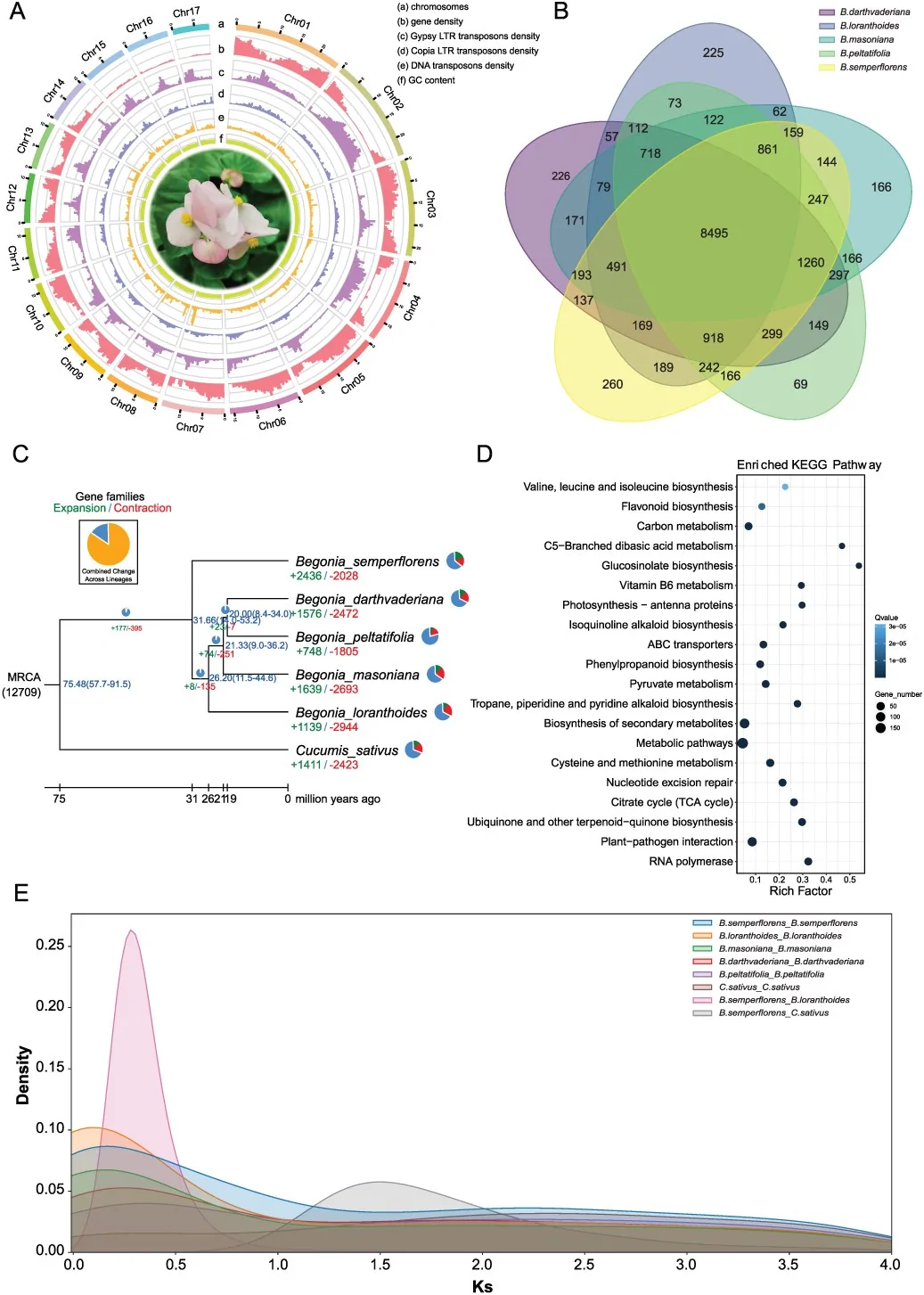
Chromosome-scale genome assembly and functional annotation of *B. semperflorens*. (A) Genome features of the *B. semperflorens* genome. (a) chromosomes, (b) gene density, (c) Gypsy LTR transposons density, (d) Copia LTR transposons density, (e) DNA transposons density, and (f) GC content. Data in panels (b–f) are based on a 500 kb sliding window. (B) Venn diagram of gene functional annotation from five databases, including NR, InterPro, KEGG, SwissProt, and TrEMBL. (C) Phylogenetic relationships and divergence of *B. semperflorens* with representative plant species. The divergence times among different plant species are labeled in the bottom. The numbers with a plus sign (+) on branches denote expanded gene families, and the numbers with a minus sign (-) indicate contracted gene families. (D) Pathway enrichment analysis of expanded gene families in *B. semperflorens*. (E) Ks distribution of *B. semperflorens* and other *Begonia* species. Peaks in interspecies comparisons indicate speciation events, while peaks in intraspecies comparisons reflect whole-genome duplication events.

We annotated 23 749 protein-coding genes. Functional assignment of these genes to public databases showed high coverage, including Nr (98.05%), TrEMBL (98.00%), InterPro (94.83%), Swiss-Prot (81.71%), Eukaryotic Orthologous Groups (KOG) (76.35%), KEGG (75.04%), and Gene Ontology (GO) (61.21%) (Table S5). A total of 116 722 590 bp of repetitive sequences were identified, accounting for 44.94% of the genome (Table S6). Long terminal repeat (LTR) retrotransposons dominated the repetitive sequences in the *B. semperflorens* genome, with Gypsy (25.58%) and Copia (9.40%) as the major components (Table S7).

OrthoFinder was employed to perform comparative clustering of protein sequences from *B. loranthoides*, *B. masoniana*, *B. peltatifolia*, and *Cucumis sativus*, yielded 17 137 orthologous gene families, 1968 of which were single-copy (Table S8 and Fig. S4). Further examination revealed that 8495 homologous gene families were conserved across all five species (Fig. 1B). The estimated divergence time of *B. semperflorens* is ~31.66 Mya, which predates that of the other *Begonia* species (Fig. 1C). Comparative analysis of gene family expansion and contraction across six plant species revealed 2437 expanded and 2029 contracted gene families in *B. semperflorens* (Fig. 1C). Among these, 91 families were significantly expanded (*P* ≤ 0.05), encompassing 582 genes, while 44 families were significantly contracted. Notably, KEGG pathway enrichment analysis of genes from significantly expanded and contracted families revealed significant enrichment in the flavonoid biosynthesis pathway, implying its potential association with evolutionary pressures in *B. semperflorens* (Fig. 1D). To further investigate the origin of this expansion, we analyzed the whole-genome duplication (WGD) events in *B. semperflorens* using the number of synonymous substitutions per synonymous site (Ks) analysis. The results indicated that *B. semperflorens* experienced only two WGD events shared with other *Begonia* species (Fig. 1E).

### *BsMYB* gene family identification and analysis

Given the potential role of the *MYB* gene family in the regulation of anthocyanins biosynthesis, we identified 209 putative *BsMYB* genes in the *B. semperflorens* genome (Table S9). These genes encode proteins ranging from 74 to 1412 amino acids in length, with molecular weights from 8664.78 to 155 752.54 Da and theoretical pI values from 4.42 to 10.21, and the encoded proteins are generally hydrophilic and mostly unstable (Table S9). Analysis of *cis*-elements in *BsMYB* promoters revealed 18 distinct types with varied functions, including low-temperature responsiveness and flavonoid biosynthetic gene regulation, implicating their potential roles in low-temperature responses and anthocyanin synthesis regulation in *B. semperflorens* (Fig. *S5*)*.*

To investigate the evolutionary relationships and potential functions of the MYB gene family in *B. semperflorens*, we constructed a phylogenetic tree containing *B. masoniana*, *B. semperflorens*, and *Arabidopsis thaliana* (Fig. S6). The results revealed a notable expansion of the S5 subfamily in *B. semperflorens* (Fig. 2A, Fig. S6). Further analysis of gene duplication events and collinearity in BsMYBs indicated significant intraspecific expansion of the S5 subfamily, with only 3 out of 21 members showing no duplication events (Fig. S7). Moreover, compared to other closely related species, this subfamily exhibited a more pronounced expansion in *B. semperflorens* (Fig. S8).

**Figure 2:**
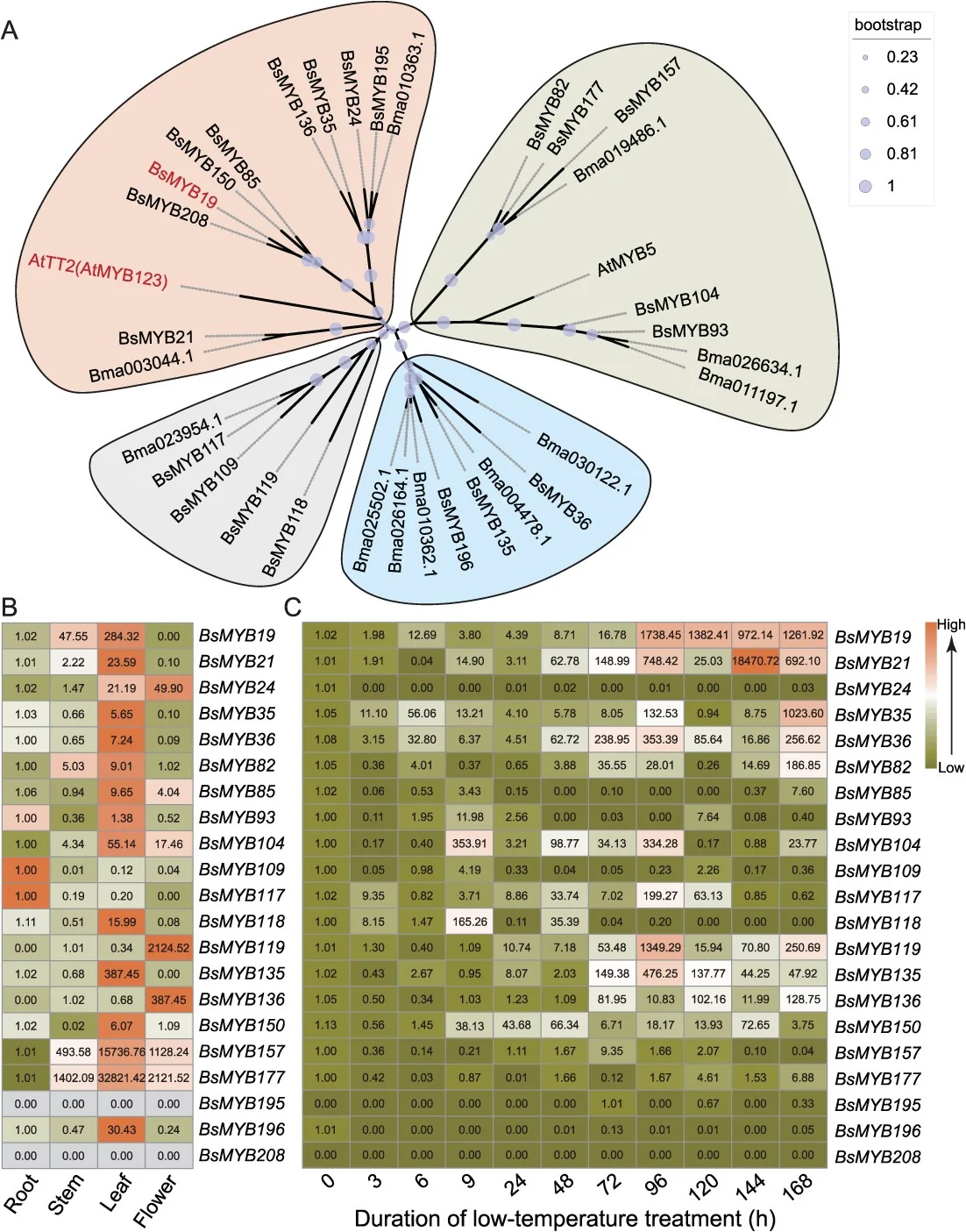
Phylogeny and expression of the BsMYB5 S5 subfamily. (A) Phylogenetic analysis of the *MYB* S5 subfamily genes from *B. semperflorens*, *B. masoniana*, and *A. thaliana*. (B) Tissue-specific expression patterns of *BsMYB* S5 subfamily genes. (C) Expression analysis of *BsMYB* S5 subfamily genes in *B. semperflorens* under low-temperature treatment (15°C/5°C). Data are shown as the mean of three biological replicates.

To further investigate the gene functions of the S5 subfamily in *B. semperflorens* and to elucidate their regulatory mechanisms, we conducted a systematic analysis of their tissue-specific expression patterns and responses to low-temperature stress using quantitative real-time PCR (qPCR) (Fig. 2B). The results revealed that *BsMYB109* and *BsMYB117* were highly expressed in roots, while the expression of most MYB S5 subfamily genes was relatively low in stems. In contrast, the majority of the genes, including *BsMYB19*, *BsMYB21*, *BsMYB35*, *BsMYB36*, *BsMYB82*, *BsMYB85*, *BsMYB93*, *BsMYB118*, *BsMYB135*, *BsMYB150*, *BsMYB155*, *BsMYB177*, and *BsMYB196*, were highly expressed in leaves. In addition, *BsMYB24*, *BsMYB135*, *BsMYB150*, and *BsMYB157* showed specifically high expression in flowers (Fig. 2B). Regarding the response to low-temperature (15°C/5°C) stress, qPCR analysis indicated that multiple genes from the S5 subfamily were up-regulated (Fig. 2C). Strikingly, the expression of *BsMYB19* increased most markedly, beginning a significant rise from the third day and surging abruptly from the fourth day (Fig. 2C). This expression pattern paralleled the trajectory of anthocyanin accumulation and was sustained at high levels thereafter, strongly suggesting that *BsMYB19* may play a regulatory role in both low-temperature (15°C/5°C) signal response and anthocyanin biosynthesis in *B. semperflorens*. To elucidate the role of *BsMYB19* in low-temperature response and anthocyanin biosynthesis regulation, this gene has been designated as *BsTT2* based on its closest *A. thaliana* homolog, which will facilitate our ongoing functional characterization (Fig. 2A).

### Role of BsTT2 in regulating anthocyanin accumulation in *B. semperflorens*

To further investigate the function of *BsTT2* in *B. semperflorens*, we conducted a series of experimental validations. Subcellular localization analysis showed that BsTT2 is localized in the nucleus (Fig. 3A). In addition, we successfully generated three transgenic *B. semperflorens* lines overexpressing BsTT2 (Fig. 3B–D). Phenotypic observation revealed that no obvious difference was observed between wild-type (WT) and *BsTT2* overexpression (OE) lines under normal temperature. However, under low temperature (15°C/5°C), the leaves of *BsTT2*-OE lines were markedly redder than those of the WT (Fig. 3B). Consistent with the phenotype, anthocyanin quantification showed no significant difference between *BsTT2*-OE lines and the WT under normal temperature, whereas under low temperature (15°C/5°C), the anthocyanin content of *BsTT2*-OE lines was significantly higher than that of the WT (Fig. 3E). In addition, analysis of the expression levels of anthocyanin biosynthesis genes revealed that BsTT2 OE significantly upregulated their expression (Fig. 3F–K).

**Figure 3:**
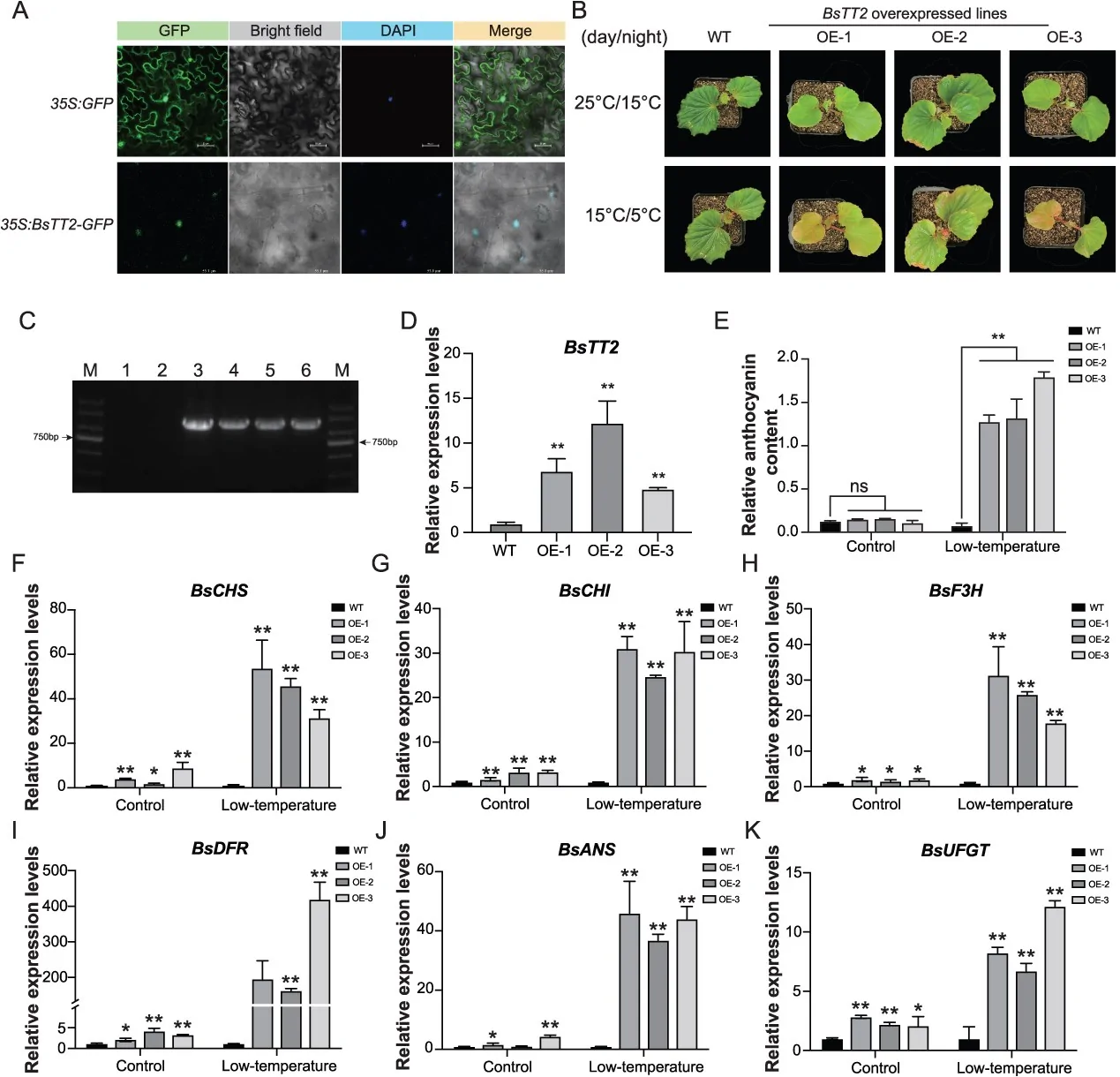
Functional analysis of the *BsTT2* gene in anthocyanin biosynthesis in *B. semperflorens*. (A) Nucleus localizations of BsTT2-GFP in tobacco epidermis cells. Channel from left to right are green fluorescent protein (GFP) fluorescence, bright field, DAPI, and merged images of tobacco leaves. (B) Phenotypic changes of *BsTT2*-overexpressed plants and wild type (WT) at normal (25°C/15°C) and low-temperature (15°C/5°C) conditions. (C) PCR verification of *BsTT2*-overexpressed lines. The electrophoretic profile displays in sequential order DL2000 DNA Marker, blank control, WT control, positive control, three independent transgenic lines (OE-1, OE-2, and OE-3), and DL2000 DNA Marker. (D) The relative expression levels of *BsTT2* in BsTT2-overexpressed lines (OE-1, OE-2, and OE-3). *Bs18S-rRNA* was used as an internal control. (E) The relative anthocyanin content in the three *BsTT2*-overexpressed lines and the WT under normal and low-temperature conditions. (F–K) Relative expression levels of the anthocyanin biosynthesis–related genes in WT and *BsTT2*-overexpressed lines under normal (25°C/15°C) and low-temperature (15°C/5°C) conditions. The error bars represent the mean ± SD of three replicates. Statistical analysis was performed using Student’s *t*-test for pairwise comparisons between wild-type and each independent overexpression line (OE-1, OE-2, and OE-3), separately (^**^P < 0.01, ^*^P < 0.05).

### BsTT2 directly binds to the *BsDFR* promoter *in vivo* and *in vitro*

To identify the target genes through which BsTT2 exerts its regulatory function, we performed Y1H assays to test its interaction with the promoters of key anthocyanin biosynthetic genes, including *BsCHS*, *BsCHI*, *BsDFR*, *BsF3H*, *BsANS*, *Bs3GT*, and *BsUFGT*. The results of Y1H showed that BsTT2 specifically binds to the promoters of *BsCHS* and *BsDFR* (Fig. 4A, Fig. S9). To further validate the functional impact of these interactions, dual-luciferase (LUC) reporter assays were conducted, and the results demonstrated BsTT2 significantly enhanced the promoter activity of *BsDFR* but not that of *BsCHS* (Fig. 4B and C, Fig. S10). The observation that BsTT2 binds the *BsCHS* promoter but does not activate it may stem from the inherent differences between the two systems. Y1H detects physical binding in a yeast context, while Dual-LUC measures functional activation in tobacco cells. An alternative explanation is that BsTT2 binds the *BsCHS* promoter but requires additional cofactors, which are absent in the Dual-LUC system, to facilitate transcriptional activation.

**Figure 4:**
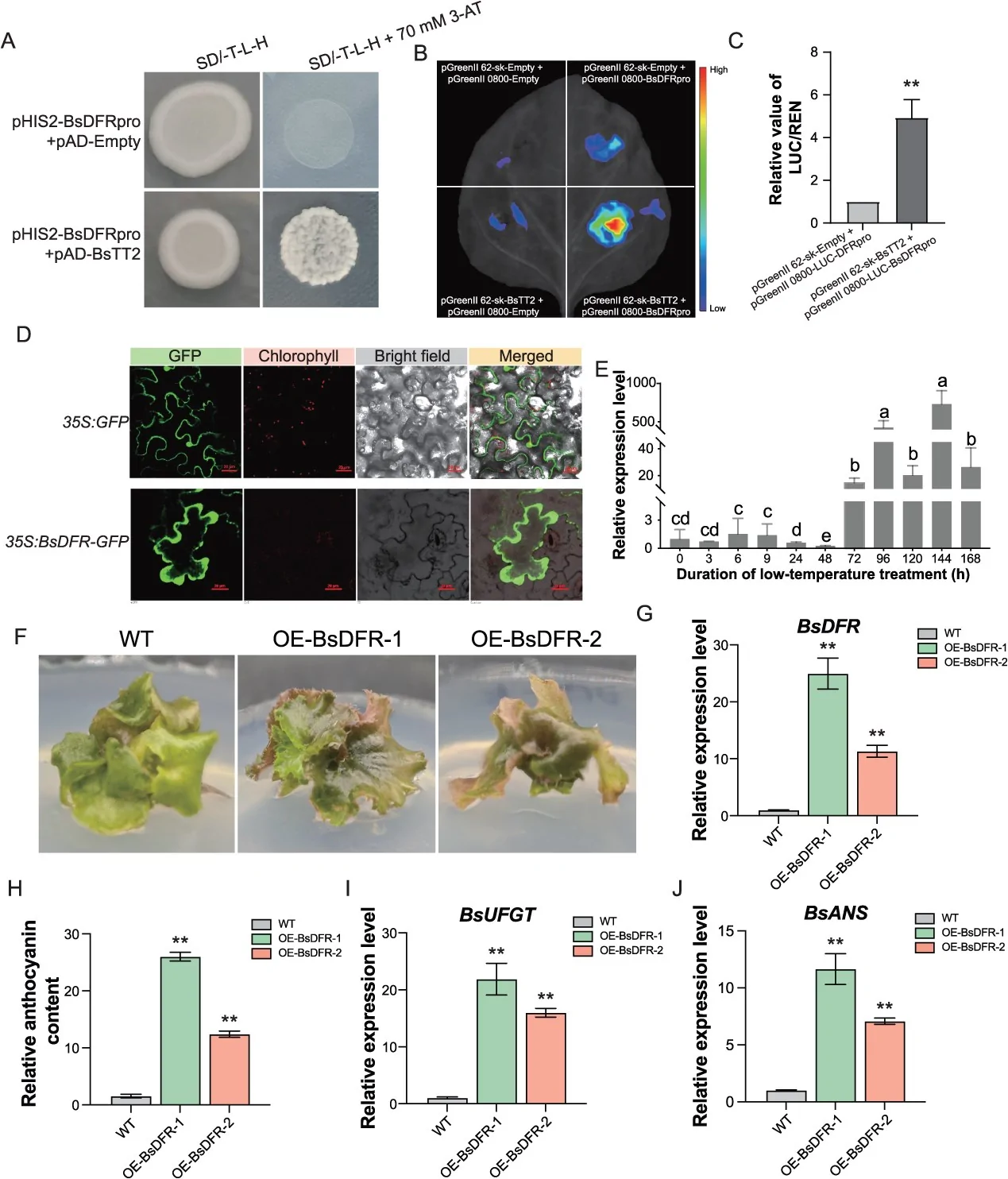
BsTT2 promotes anthocyanin biosynthesis by binding and activating the *BsDFR* promoter. (A) Physical interactions of BsTT2 with the *BsDFR* promoter in yeast one-hybrid (Y1H) assays. (B) Representative bioluminescence images of transcriptional activity assay in *N. benthamiana* leaves. (C) LUC/REN ratio in the dual-LUC assays. The error bars represent the mean ± SD of three replicates. Asterisk above the bars indicate statistically significant differences (Student’s *t*-test, ^**^P < 0.01, ^*^P < 0.05). (D) The localization of the BsDFR-GFP protein in the *N. benthamiana* leaves. (E) Expression analysis of *BsDFR* genes in *B. semperflorens* under low-temperature (15°C/5°C) treatment. The error bars represent the mean ± SD of three replicates. Different letters above the histogram indicate significant differences among different treatments (*P* < 0.05) using one-way ANOVA. (F) Phenotypic changes of *BsDFR*-overexpressed plants (OE-BsDFR-1 and OE-BsDFR-2) and wild type (WT) at normal (25°C/15°C) condition. (G) The relative expression levels of *BsDFR* in *BsDFR*-overexpressed lines (OE-BsDFR-1 and OE-BsDFR-2). *Bs18S-rRNA* was used as an internal control. (H) The relative anthocyanin content in *BsDFR*-overexpressed lines and the WT under normal condition. (I, J) Relative expression levels of the anthocyanin biosynthesis-related genes in WT and *BsDFR*-overexpressed lines under normal condition. The error bars represent the mean ± SD of three replicates. Statistical analysis was performed using Student’s *t*-test for pairwise comparisons between wild-type and each independent overexpression line (OE-BsDFR-1 and OE-BsDFR-2), separately (^**^P < 0.01, ^*^P < 0.05).

### Functional analysis of BsDFR in anthocyanin accumulation in apple

To further elucidate the function of *BsDFR* in anthocyanin biosynthesis, we characterized the *BsDFR* gene and expressed it transiently in apple peels. Subcellular localization analysis revealed that the BsDFR is localized in the cytoplasm (Fig. 4D). In addition, we analyzed the expression pattern of *BsDFR* in *B. semperflorens* under low-temperature treatment and found that its transcript level increased significantly after 72 h of treatment and remained stably high (Fig. 4E). Notably, this expression trend was nearly identical to that of *BsTT2* in *B. semperflorens*. Transient OE of *BsDFR* in apple peel promoted anthocyanin accumulation (Fig. S11). To further verify the endogenous function of *BsDFR* in *B. semperflorens*, we generated *BsDFR*-overexpressing transgenic lines (Fig. 4F and G). Compared with the WT, the OE lines exhibited significantly enhanced anthocyanin pigmentation (Fig. 4F) and elevated anthocyanin content (Fig. 4H), accompanied by upregulated expression of key anthocyanin biosynthetic genes (*BsUFGT* and *BsANS*) (Fig. 4I–K). These results indicate that *BsDFR* contribute to anthocyanin biosynthesis in *B. semperflorens*.

### BsTT2 interacts with BsAlfin2 both *in vitro* and *in vivo*

To further investigate the mechanism of BsTT2, we performed a Y2H screen using BsTT2 as bait and identified two candidate interacting proteins. Among them, a transcription factor (g14039.t1) belonging to the *Alfin* gene family was selected for further study, as previous studies have reported that *Alfin* gene family members (*GmAlfin09*) are involved in the ROS signaling pathway, which aligns with the focus of this study. In addition, based on phylogenetic analysis, this gene was designated as *BsAlfin2* (Fig. S12). In directed Y2H assays, yeast cells co-expressing BsTT2 and BsAlfin2 exhibited robust growth on selective medium and developed blue colonies in the presence of X-*α*-gal, whereas negative controls showed no growth, demonstrating a specific interaction between BsTT2 and BsAlfin2 in the yeast system (Fig. 5A). To further validate this interaction *in vivo*, we conducted a bimolecular fluorescence complementation (BiFC) assay, and results showed that strong YFP fluorescence was detected in the nucleus of tobacco leaf cells co-expressing BsTT2-pCAMBIA1300-nYFP and BsAlfin2-pCAMBIA1300-cYFP (Fig. 5B). In addition, a pull-down assay confirmed that BsAlfin2 directly binds to BsTT2 (Fig. 5C).

**Figure 5:**
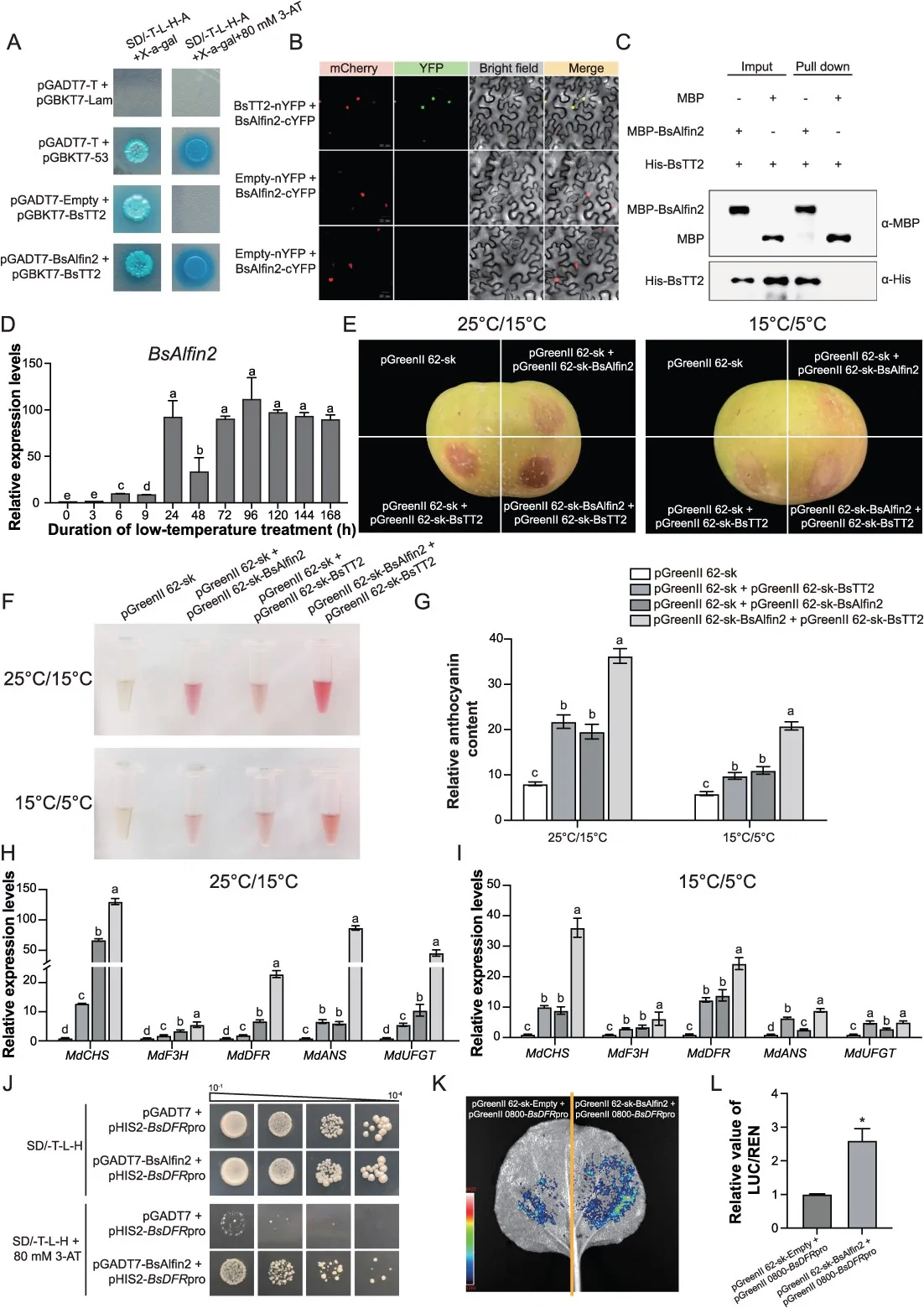
BsTT2 interacts with BsAlfin2 to synergistically promote anthocyanin biosynthesis. (A) Verification of the activation ability of transcription factor BsAlfin2 by using yeast strain Y2HGold. pGBKT7-53 and pGADT7-T was the positive control, and pGBKT7-lam and pGADT7-T was the negative control, pGBKT7-BsTT2 and pGADT7-Empty was the empty control. The results were judged by the growth of the colony on a plate with medium SD/–Trp/–Leu/–His/Ade + 80 mM 3-AT + X-α-Gal. (B) Bimolecular fluorescence complementation of BsTT2 and BsAlfin2. (C) Pull-down assay confirmed a direct physical interaction between BsTT2 and BsAlfin2. (D) Expression analysis of *BsAlfin2* genes in *B. semperflorens* under low-temperature treatment. (E, F) Phenotypic analysis of the transient transformation of *BsTT2, BsAlfin2,* and *BsTT2 + BsAlfin2* co-transformation in apple*.* (G) Relative anthocyanin content in apple fruits subjected to transient transformation with different gene constructs under normal (25°C/15°C) and low-temperature (15°C/5°C) conditions. (H, I) The expression levels of anthocyanin biosynthesis genes in apple fruits subjected to transient transformation with different gene constructs under normal (25°C/15°C) and low-temperature (15°C/5°C) conditions. The error bars represent the mean ± SD of three replicates. Different letters above the histogram indicate significant differences among different treatments (*P* < 0.05) using one-way ANOVA. (J) Physical interactions of BsAlfin2 with the *BsDFR* promoter in Y1H assays. (K) Representative bioluminescence images of transcriptional activity assay in *N. benthamiana* leaves. (L) LUC/REN ratio in the dual-LUC assays. The data are the mean ± SD of three replicates. Asterisk above the bars indicate statistically significant differences (Student’s *t*-test, ^**^P < 0.01, ^*^P < 0.05).

### BsTT2 and BsAlfin2 synergistically promote anthocyanin biosynthesis

We further examined the expression pattern of *BsAlfin2* under low-temperature treatment. The results showed that *BsAlfin2* expression levels was significantly upregulated after 6 h of low-temperature exposure and remained at elevated levels throughout the treatment period (Fig. 5D). Using a transient expression system in apple, we found that under both normal and low-temperature conditions, apple peel overexpressing *BsAlfin2* exhibited enhanced red pigmentation and accumulated more pigment compared to the control (Fig. 5E-F and Fig. S13). Consistent with these phenotypic observations, anthocyanin content was significantly higher in *BsAlfin2*-overexpressing tissues under both temperature regimes (Fig. 5G). Furthermore, the expression levels of key anthocyanin biosynthesis genes were markedly upregulated in the transgenic tissues relative to control samples at both temperatures (Fig. 5H and I). Notably, co-transformation of *BsTT2* and *BsAlfin2* promoted anthocyanin synthesis more effectively than transformation with *BsTT2* or *BsAlfin2* alone (Fig. 5E–I), indicating a synergistic interaction between these two regulators in enhancing anthocyanin accumulation.

To further elucidate the molecular mechanism by which BsAlfin2 promotes anthocyanin synthesis, we investigated its relationship with BsDFR using Y1H and LUC assays. The results demonstrated that BsAlfin2 directly binds to the *BsDFR* promoter and significantly enhances its transcriptional activity (Fig. 5J–L).

### BsAlfin2 mediates ROS-dependent nuclear translocation to enhance *BsDFR* activation under low temperature

To further investigate the regulatory relationship between ROS signaling and anthocyanin biosynthesis in *B. semperflorens*, we examined the expression levels of *BsTT2* and *BsAlfin2* following H_2_O_2_ treatment. Both genes showed significant up-regulation at 24 h after H_2_O_2_ application (Fig. 6A and B), indicating their responsiveness to ROS signaling.

**Figure 6:**
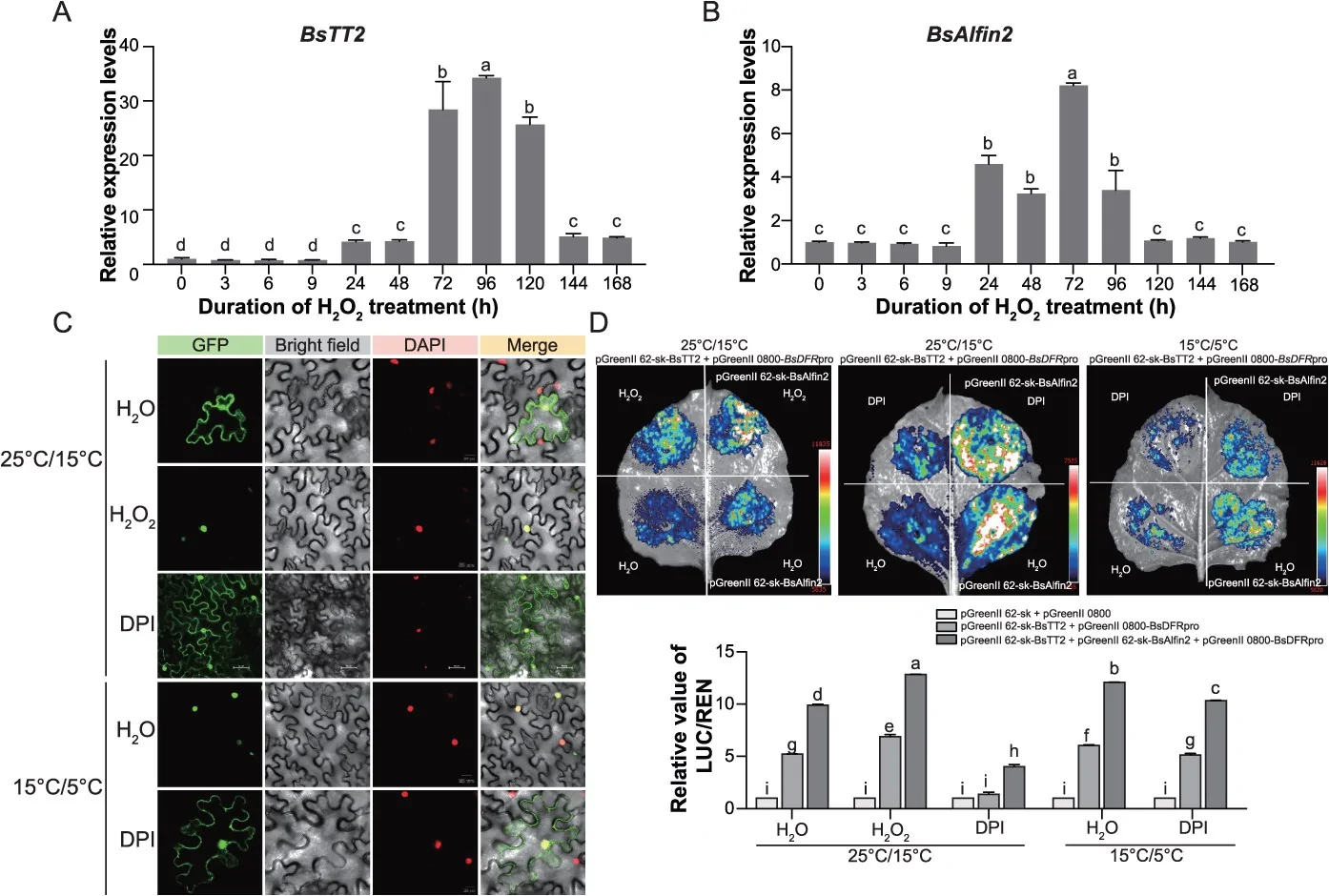
The BsAlfin2-BsTT2-BsDFR regulatory module for anthocyanin biosynthesis is ROS-dependent. (A, B) The qPCR analysis of the *BsTT2* and *BsAlfin2* under H_2_O_2_ treatment. Different lowercase letters above the bars indicate statistically significant differences (*P* < 0.05) using one-way ANOVA. (C) Localization of BsAlfin2 in *N. benthamiana* cells under different treatment. (D) LUC/REN ratio in the dual-LUC assays. The treatments were water, H_2_O_2_, or DPI spraying at 25°C/15°C, water or DPI spraying at 15°C/5°C. The data are the mean ± SD of three replicates. Different letters above the histogram indicate significant differences among different treatments (*P* < 0.05) using one-way ANOVA.

We next employed a tobacco transient expression system to investigate the subcellular localization dynamics of BsAlfin2 in response to environmental signals. Under normal conditions, BsAlfin2 localized to both the nucleus and cytoplasm (Fig. 6C). However, low-temperature treatment induced pronounced nuclear accumulation of BsAlfin2 (Fig. 6C). This nuclear translocation was similarly triggered by H_2_O_2_ treatment, while application of the ROS inhibitor diphenyleneiodonium (DPI) under low-temperature conditions restored cytoplasmic localization (Fig. 6C). These results demonstrate that low temperature promotes nuclear translocation of BsAlfin2 through an H_2_O_2_-dependent mechanism (Fig. 6C). In addition, western blot analysis revealed that both exogenous H_2_O_2_ treatment and low-temperature treatment significantly increased the abundance of BsAlfin2 protein (Fig. S14).

To further elucidate the roles of *BsTT2* and *BsAlfin2* in mediating the regulatory relationship between ROS signaling and anthocyanin biosynthesis in *B. semperflorens*, we examined the effects of low temperature and ROS on *BsDFR* promoter activity. Compared to BsTT2 expression alone, co-expression of BsTT2 and BsAlfin2 synergistically enhanced BsDFR promoter activity under both normal (25°C/15°C) and low-temperature (15°C/5°C) conditions (Fig. 6D). Notably, H_2_O_2_ treatment significantly increased *BsDFR* promoter activity at both 25°C/15°C and 15°C/5°C, whereas application of the ROS inhibitor DPI at 15°C/5°C substantially suppressed this activation (Fig. 6D). These results demonstrated that ROS generated under low-temperature conditions promote BsDFR transcriptional activation by enhancing the regulatory capacity of its transcription factors.

## Discussion

In this study, we established the first chromosome-level genome assembly for *B. semperflorens* through comprehensive genomic characterization and *de novo* assembly. Our analysis systematically revealed fundamental aspects of genome architecture, evolutionary history, and functional divergence within key gene families. Furthermore, we proposed and experimentally validated a novel BsAlfin2/BsTT2-BsDFR regulatory module that mediates anthocyanin biosynthesis activation in response to low-temperature-induced ROS signaling.

### The assembly of the *B. semperflorens* genome has unveiled a WGD event unique to the Neotropical clade

In this study, we successfully assembled a reference genome for *B. semperflorens* with a total size of 259.68 Mb and a scaffold N50 of 14.43 Mb, demonstrating high assembly continuity (Table S3). In addition, compared with other reported *Begonia* species, including *B. loranthoides*, *B. masoniana*, *B. darthvaderiana*, and *B. peltatifolia*, *B. semperflorens* exhibits relatively lower proportions of both total repetitive sequences and LTR-Gypsy elements, suggesting that its genome is more compact with fewer active transposable element (TE) insertion events [21]. This more compact genome may contribute to maintaining an efficient gene expression regulatory network and reducing transposon-mediated interference with functional genes, potentially underpinning its broad environmental adaptability and rapid growth characteristics. In terms of evolutionary divergence, *B. semperflorens* represents an early-diverging lineage compared with other species within the genus *Begonia* (Fig. 2C), which is consistent with its relatively compact genome and lower repeat sequence content. Later-diverging *Begonia* species may have experienced more active TE expansion and genomic rearrangements, potentially contributing to their greater morphological and metabolic diversity [21]. More importantly, we found that the flavonoid biosynthesis pathway was significantly enriched among the expanded gene families (Fig. 2D), suggesting that the expansion of this pathway may provide a genomic basis for the rich leaf color variation observed in *B. semperflorens* [22].

Previous genomic studies, such as in opium poppy, have established that WGD events can promote chromosomal rearrangements and enhance secondary metabolism through nonrandom retention of key metabolic genes [23]. Similarly, WGD has been implicated in the evolutionary success of Asteraceae, the largest angiosperm family [24]. These events are generally categorized as autopolyploid or allopolyploid, with autopolyploidy frequently associated with environmental adaptation, as demonstrated in ancient plant lineages [25]. Alternatively, some WGDs may originate from hybridization, a pattern observed in the closely related Gesneriaceae family [26]. Nevertheless, elucidating the precise mechanism remains challenging due to the complex interplay between historical climate fluctuations and polyploid establishment. In our study, combined with Ks distribution and gene family evolution analyses, we further investigated the WGD events in *B. semperflorens*. The Ks analysis revealed two key polyploidization events in *B. semperflorens* (Fig. 1E). The peak at ~1.5 corresponds to the core eudicot-shared hexaploidization event [27], while the peak at ~0.5 suggests a lineage-specific WGD event in *Begonia*. These two events provided the genetic basis for the expansion of the MYB gene family, with the *Begonia*-specific WGD further increasing the copy number of paralogous MYB genes and laying the foundation for subsequent functional divergence. Compared with other *Begonia* species, although *B. semperflorens* shares this ancestral WGD, it exhibits a unique pattern of gene family expansion and contraction (Fig. 1C), which may be associated with its species-specific environmental adaptations and the functional specialization of the BsMYB family.

MYB transcription factors often respond to environmental changes by activating or regulating metabolic pathways to enhance plant resistance. Analysis of the MYB gene family in the *B. semperflorens* genome identified 320 members, significantly more than in other *Begonia* varieties. This increase may be attributed to the WGD event. Analysis of the Ka/Ks values for the MYB gene family showed that nearly all values were <1, indicating purifying selection and the removal of redundant members generated during MYB family expansion. This clearance suggests functional redundancy, with some redundant genes undergoing retention, loss, silencing, functional diversification, or subfunctionalization. These gene alterations likely result in a more complex regulatory network, potentially enhancing the stress resistance of *B. semperflorens* [28].

### BsTT2 promotes anthocyanin accumulation by binding and activating the *BsDFR* promoter in *B. semperflorens*

MYB transcription factors involved in anthocyanin biosynthesis often function as critical regulatory hubs within signaling networks [29]. In grape, VvMYBA1 and VvMYB3 form an activation repression system in which VvMYB3 serves as a key node to fine-tune anthocyanin biosynthesis [30]. In this study, we systematically analyzed the *BsMYB* gene family in *B. semperflorens* and identified a unique S5 subfamily within the R2R3-MYB group. The S5 subfamily member is known to be involved in anthocyanin biosynthesis. For example, in apple, the MdMYB305-bHLH33-MYB10 module coordinates sugar metabolism and anthocyanin accumulation, with MdMYB305 acting as a central regulatory node [31]. Comparative evolutionary analysis revealed that this subfamily exhibits both enhanced branch diversification and an increased number of genes compared to *A. thaliana* and other *Begonia* species (Fig. S6), suggesting the S5 subfamily in *B. semperflorens* may function through a similar pathway, though its expansion implies a more complex regulatory network.

Gene expression patterns are closely linked to its function [32]. Tissue-specific expression and low-temperature treatment analyses revealed that *BsMYB19* (*BsTT2*) is not expressed in floral tissues but shows sustained high expression under low-temperature conditions, consistent with the observed anthocyanin accumulation in plants under low-temperature stress (Fig. 2B and C, Fig. S1). These results suggest that *BsTT2* is a key candidate regulator of low-temperature-induced anthocyanin biosynthesis in *B. semperflorens*. Consistently, functional validation through transgenic assays demonstrated that *BsTT2*-overexpressing plants exhibited accelerated and enhanced anthocyanin accumulation under low-temperature stress, confirming its direct role in promoting anthocyanin biosynthesis (Fig. 3B–E). In addition, these results indicate that BsTT2 acts as a low-temperature-enhanced regulator of anthocyanin biosynthesis in *B. semperflorens*. This functional characteristic is similar from known temperature-responsive MYB factors in other plants. For example, MdMYB23, as a key regulator of proanthocyanidin biosynthesis, to promote proanthocyanidin accumulation and ROS scavenging under cold stress in apple [33]. However, it should be noted that other parallel regulatory pathways likely exist in *B. semperflorens*. In addition to BsTT2, multiple other BsMYB family members, including *BsMYB21*, *BsMYB35*, *BsMYB119*, and *BsMYB135*, are significantly upregulated under low-temperature treatment (Fig. 2C). These MYB genes may exhibit functional redundancy or division of labor, differentially regulating anthocyanin biosynthesis in distinct tissues or under different stress conditions. Furthermore, given that anthocyanin biosynthesis is primarily regulated by the MBW complex in many plant species [34], we speculate that a similar regulatory mechanism may exist in *B. semperflorens*. In addition to BsTT2, other upregulated BsMYBs may form MBW complexes with specific bHLH proteins to regulate anthocyanin biosynthesis, a possibility that requires further investigation.

As a transcription factor, BsTT2 functions by regulating the promoters of downstream genes. DFR, a key structural gene in anthocyanin biosynthesis, has been extensively characterized across diverse plant species and frequently functions as a terminal node in transcriptional regulatory networks [35]. In this study, we demonstrated that BsTT2 specifically binds to and activates the *BsDFR* promoter, revealing that BsTT2 enhances both anthocyanin biosynthesis and stress resistance in *B. semperflorens* by mediating low-temperature stress responses and upregulating *BsDFR* expression.

### BsTT2 and BsAlfin2 synergistically regulate anthocyanin biosynthesis in *B. semperflorens*

Plant traits are often precisely regulated by multi-gene regulatory networks. To further elucidate the regulatory mechanism underlying anthocyanin biosynthesis, we screened and identified BsAlfin2 as a transcription factor that interacts with BsTT2 (Fig. 5C–E). Studies on Alfin-like transcription factors are limited, and have been conducted in only a few plant species [36–38]. Alfin-like transcription factors are known to play roles in developmental processes such as seed germination, root development, and root-hair elongation [39]. However, their sensitivity to environmental stress and specific molecular mechanisms remains underexplored [40]. Here, BsAlfin2 functioned as a low-temperature inducible transcriptional regulator that promoted anthocyanin accumulation, with co-expression of BsAlfin2 and BsTT2 resulting in significantly enhanced anthocyanin production in apple (Fig. 5E–I). Furthermore, we confirmed that BsAlfin2 activates *BsDFR* expression through direct binding to its promoter (Fig. 5J–L). These results demonstrated that BsAlfin2 interacted with BsTT2 and activated *BsDFR* expression by binding to its promoter, thereby synergistically promoting anthocyanin accumulation. Nevertheless, given the species differences between apple (Rosaceae) and *B. semperflorens* (Begoniaceae), the apple peel transient system should be regarded as a preliminary heterologous validation platform. Future establishment of more sophisticated genetic tools in *B. semperflorens* will be required to further dissect the regulatory mechanisms of BsTT2 and BsAlfin2 in their natural context.

### The BsAlfin2-BsTT2-BsDFR module regulates anthocyanin biosynthesis in *B. semperflorens* in a ROS-dependent manner

Recent studies indicated that *Alfin* gene family members respond to abiotic and biotic stresses by regulating ROS-related genes [19, 31, 41]. For example, MdAL4 binds to the promoters of ROS-scavenging genes and reduces ROS accumulation under drought stress in apple [42]. In contrast, AL7 enhances ROS accumulation during immune responses by modulating the expression of downstream ROS-scavenging genes in *N. benthamiana* [31]. This comparison highlights the functional divergence of AL-family proteins in regulating ROS homeostasis. In our study, *BsAlfin2* and *BsTT2* showed similarly significant upregulation in response to H_2_O_2_ treatment (Fig. 6A and B), indicating their functional association with ROS signaling.

Subcellular localization revealed that BsAlfin2 is distributed in both the cytoplasm and nucleus (Fig. 6C, Fig. S15), whereas previously reported Alfin family members are localized and function solely in the nucleus [18, 19]. To investigate the mechanism underlying its cytoplasmic localization, we subjected BsAlfin2-EGFP-transformed tobacco leaves to low-temperature treatment. And we observed the disappearance of cytoplasmic localization without changes in total protein levels (Fig. 6C, Fig. S14), indicating that BsAlfin2 undergoes nuclear translocation rather than degradation. Similar nuclear translocations have been observed for antifreeze-related genes like *NLP7* and *CPK28* in rice under low-temperature treatment [43]. We next investigated whether ROS signaling mediate this process. Treatment with H_2_O_2_ at 25°C fully recapitulated the low-temperature-induced nuclear accumulation of BsAlfin2 (Fig. 6C). Conversely, when leaves were treated with the ROS scavenger DPI under low-temperature conditions, nuclear translocation was abolished (Fig. 6C). Above results established that low-temperature triggers BsAlfin2 nuclear import through an ROS-dependent mechanism. However, the detailed molecular mechanism underlying BsAlfin2 nuclear translocation remains to be deeply elucidated. Several possible mechanisms may be involved. First, low-temperature-induced ROS signaling may promote phosphorylation of BsAlfin2, thereby altering its affinity for nuclear transport receptors and facilitating nuclear import. Second, BsAlfin2 may interact with specific nuclear transport proteins and enter the nucleus via active transport. Third, low temperature or ROS signaling may influence the permeability or conformational state of the nuclear pore complex, indirectly affecting the nucleocytoplasmic distribution of BsAlfin2. These hypotheses require systematic validation through approaches such as phosphorylation site mutagenesis, nuclear transport receptor interaction analyses, and chemical inhibitors, which represent key directions for our future research.

To further investigate the role of the interaction between nuclear-translocated BsAlfin2 and BsTT2 in regulating anthocyanin synthesis, we performed co-transformation assays in tobacco leaves. We found that the co-expression of BsAlfin2 and BsTT2 significantly up-regulated *BsDFR* expression. Furthermore, H_2_O_2_ treatment further enhanced *BsDFR* promoter activity (Fig. 6D). Our previous studies have identified ROS as a key inducer of low-temperature-induced anthocyanin biosynthesis in *B. semperflorens* [2, 16]. Building on this knowledge, we further demonstrated that ROS generated under low-temperature treatment promote the nuclear translocation of BsAlfin2. The nuclear BsAlfin2 then interacts with BsTT2 to form a transcriptional complex, which activates the *BsDFR* promoter and ultimately improves the efficiency of anthocyanin biosynthesis.

## Conclusion

We successfully assembled a high-quality chromosome-level genome of *B. semperflorens*, which serves as a valuable genomic resource for future research. More importantly, we identified and functionally characterized a key regulatory module, BsAlfin2/BsTT2-BsDFR, that plays a central role in anthocyanin biosynthesis (Fig. 7). In this module, BsTT2 directly activated *BsDFR* expression by binding to its promoter. Meanwhile, under low-temperature-induced ROS signaling, BsAlfin2 translocates into the nucleus and interacts with BsTT2, which synergistically enhances the transcriptional activation of *BsDFR* and thereby promotes anthocyanin accumulation. This module not only elucidates a key molecular pathway regulating leaf color in *B. semperflorens* but also provides the first evidence linking low temperature, ROS signaling, and anthocyanin biosynthesis in this species. These results provide a solid molecular target and conceptual framework for horticultural breeding targeting leaf color improvement and enhanced stress resistance.

**Figure 7:**
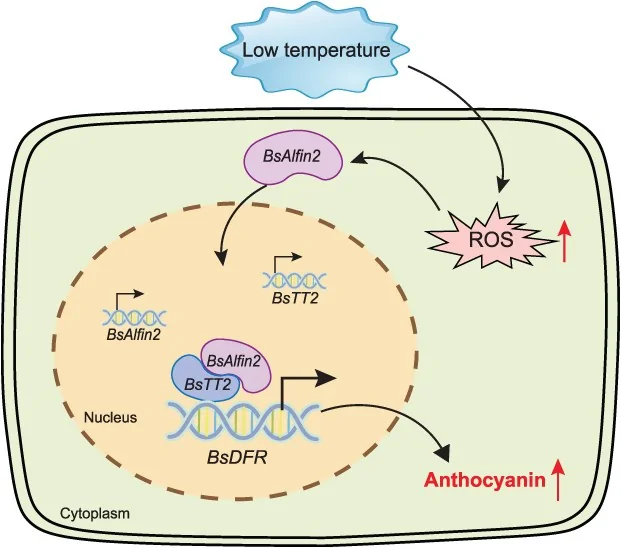
A hypothetical model illustrating the regulatory mechanism of the BsAlfin2/BsTT2-BsDFR module in anthocyanin biosynthesis in *B. semperflorens*. Under low-temperature induced ROS signaling, BsAlfin2 translocates into the nucleus and interacts with BsTT2, synergistically enhancing the transcriptional activation of *BsDFR* and thereby promoting anthocyanin biosynthesis in *B. semperflorens*.

## Materials and methods

### Plant materials and treatments

Seeds of *B. semperflorens* ‘Super Olympics’ were cultivated in the laboratory of the College of Landscape Architecture and Art, Henan Agricultural University. Plants were grown in a growth chamber set to a 14/10-h dark/light cycle with corresponding temperatures of 25°C during the dark phase and 15°C during the light phase, under 65% relative humidity (RH) and a light intensity of 200 μmol m^2^ s^−1^ during the light period. Thirty healthy plants were subjected to a low-temperature treatment at 15°C (day) and 5°C (night) with an RH of 65%. Three biological replicates were performed.

### Genome sequencing

Leaf tissues from a single individual of *B. semperflorens* were collected for genomic DNA extraction using the cetyltrimethylammonium bromide method. Whole-genome sequencing was conducted using the MGI DNA Library Universal Kit according to the manufacturer’s instructions. After library qualification, sequencing was carried out on the MGI-SEQ 2000 platform. For PacBio HiFi sequencing, the SMRTbell Express Template Prep Kit 2.0 (Pacific Biosciences) was used to construct the SMRTbell HiFi library. Following library qualification, sequencing was performed on the PacBio Revio platform.

### Genome survey, assembly, and assessment

The *k*-mer frequency distribution for *k*-values ranging from 17 to 31 was first calculated using Jellyfish v2.2.10 [44]. Subsequently, the genome characteristics were estimated by fitting the *k*-mer spectra with GenomeScope [45]. For PacBio sequencing, raw data were processed using SOAPnuke (v1.5.3) to filtering out low-quality reads, adapter sequences, and short fragments, yielding 27.87 Gb of high-quality subreads (Table S1). The final genome assembly was generated from these subreads using hifiasm [46]. After genome assembly, the assembled scaffolds were aligned against plant mitochondrial and chloroplast reference genomes (NCBI RefSeq) using BLASTn to filter mitochondrial/chloroplast sequences. Potential contamination was assessed using kraken2 for taxonomic classification of raw reads, combined with BLASTn against the NC NT database and protein sequence annotation against UniProt/Cluster of Orthologous Groups (COG)/KEGG to detect and remove exogenous contaminants. We primarily employed the 3D-DNA pipeline to cluster, order, and orient the genomic sequences, followed by comprehensive evaluation of the assembly results. The contig-level genome assembly obtained in the previous step was upgraded to chromosome-level using Hi-C data assisted by Juicer and 3D-DNA [47]. The assembly quality was assessed using BUSCO with the embryophyta_odb12 dataset [48].

### Genome annotation

The comprehensive annotation of repetitive sequences in the *B. semperflorens* genome was performed through an integrated approach combining homology-based and *de novo* prediction methods. Tandem repeats were identified using Tandem Repeats Finder v4.09 with default parameters [49]. For TE annotation, we employed a dual strategy: homology-based screening was conducted using RepeatMasker v4.1.2 and RepeatProteinMask v4.0.7 against the RepBase database to identify known repetitive elements [50]. Simultaneously, a *de novo* repeat library was constructed using LTRharvest from GENOMEtools v1.5.10 and RepeatModeler v2.0.3, followed by annotation of novel repetitive elements using RepeatMasker [51].

Functional annotation of the predicted protein-coding genes was performed through a comprehensive multi-database approach. First, BLASTP alignments were conducted against public databases including NCBI nonredundant (Nr) and Swiss-Prot using an *E*-value cutoff of 1 × 10^−5^. Protein domains and functional sites were subsequently identified using InterProScan (version 4.8) by searching against all member databases. Additionally, eggNOG-mapper (v2) was employed to assign COG categories, GO terms, and KEGG pathway annotations [52]. This integrated strategy ensured comprehensive functional characterization of the predicted gene repertoire.

### Phylogenetic tree construction

Gene family clustering was performed using OrthoFinder v2.5.5 [53] with appropriate species (Schedule S1). Protein sequences were aligned using MUSCLE v5.1 [54] and trimmed with TrimAL v1.4.1 [55]. A supermatrix was generated, and the phylogenetic tree was constructed using IQ-TREE v2.2.2.7 [56]. Fossil time data from TimeTree [57] were used for calibration. Branch lengths were estimated using BASEML from the PAML package [58]. The quality was assessed with Tracer v1.7.2 [59] to ensure a valid sample size >200. Gene family expansion and contraction analyses were conducted using CAFE v5.0.0 [60] based on estimated species differentiation times.

### Genome-wide identification of *MYB* family genes from *B. semperflorens* genome

The Pfam database [61] was used to predict genome-wide domains, filtered by a threshold of 1e^−5^. Genes containing ‘PF00249’ were identified as members of the MYB transcription factor family. Motif analysis was conducted using MEME v5.0.5 [62], and MYB gene family correlation analysis and mapping were performed with TBtools [63].

### Quantitative real-time PCR analysis

Total RNA using FastPure Universal Plant Total RNA Isolation Kit (Vazyme, China). DNA-free RNA was used to synthesize the first strand of cDNA with the HiScript III 1st Strand cDNA Synthesis Kit (Vazyme, China). Primers for qPCR are shown in Table S11. qPCR was performed in a CFX384 Touch Real-Time PCR Detection System (Biorad, USA) with ChamQ Universal SYBR qPCR Master Mix (Vazyme, China), and the procedure followed the reagent and instrument specifications. The relative expression level of genes was calculated by 2^−ΔΔCT^ method [64] and normalized by 18 s rRNA, a reference gene in *B. semperflorens*, and AtActin, a reference gene in *Malus domestica*, respectively.

### Gene cloning and vector construction

The coding sequence (CDS) of *BsMYB* and *BsDFR* gene, and *BsDFR*, *Bs3GT*, *BsANS*, *BsCHI*, *BsCHS*, *BsF3H*, and *BsUFGT* promoter was amplified by PCR using KOD One™ PCR Master Mix -Blue- (TOYOBO, Japan). Primers are shown in Table S10. *BsTT2* were cloned into the pGADT7 vector (pAD-BsTT2), and the *BsDFR* promoter fragment was cloned into the pHIS2 vector using ClonExpress® II One Step Cloning Kit (Vazyme, China). The *BsCHS*, *BsCHI*, *BsF3H*, *BsANS*, *Bs3GT*, and *BsUFGT* promoter was cloned into the pAbAi vector (promoters-pAbAi) for yeast one-hybrid experiments. *BsTT2* and *BsAlfin2* were cloned into the pGreenII 62sk vector, while the *BsDFR*, *BsCHS* promoter fragment was cloned into the pGreenII 0800 vector for dual-LUC experiments. *BsTT2* and *BsAlfin2* were cloned into the pCanG vector [65] for OE and subcellular localization. pGBKT7-BsTT2 was constructed for yeast two-hybrid (Y2H) screening. *BsTT2* and *BsAlfin2* were cloned into pCAMBIA1300-35S-nYFP and pCAMBIA1300-35S-cYFP for BiFC assays.

### Subcellular localization

The constructs BsTT2-pCanG, BsDFR-pCanG, and BsAlfin2-pCanG were individually transformed into *Nicotiana benthamiana* leaves by *Agrobacterium*-mediated transformation [66]. The lower epidermis of tobacco leaves was examined under confocal microscopy to confirm their subcellular localization.

In addition, *N. benthamiana* leaves that had been successfully transformed with the BsAlfin2-pCanG construct were subjected to five different treatments. Under normal conditions, leaves were sprayed with water, 5 mM H_2_O_2_, or 0.01 mM DPI, respectively. Under low-temperature conditions, leaves were sprayed with either water or 0.01 mM DPI. After 6 h of treatment, the subcellular localization of BsAlfin2 in *N. benthamiana* epidermal cells was observed under a laser scanning confocal microscope.

### Generation of *BsTT2*-overexpressed *B. semperflorens*

Leaves of *B. semperflorens* were cut into uniform pieces and pre-cultured for 1 day in MS medium supplemented with 0.5 mg l^−1^ 6-BA and 0.12 mg l^−1^ NAA. After preculture, the leaves were infected with an *Agrobacterium* suspension (OD600 = 0.7) in MS medium without sucrose or agar for 8 m, followed by co-culture in MS medium supplemented with 0.5 mg l^−1^ 6-BA, 0.12 mg l^−1^ NAA, and 100 μM As for 4 days. Transition cultures were maintained for 7 days in MS medium containing 0.5 mg l^−1^ 6-BA, 0.12 mg l^−1^ NAA, and 400 mg l^−1^ Carb. Screening was performed on MS medium with 0.5 mg l^−1^ 6-BA, 0.12 mg l^−1^ NAA, 400 mg l^−1^ Carb, and 25 mg l^−1^ Kan. The rooting medium consisted of 1/2 MS medium supplemented with 0.1 mg l^−1^ NAA, 400 mg l^−1^ Carb, and 25 mg l^−1^ Kan [67]. Positive plants were identified by detecting *BsTT2* gene expression and confirming the presence of the target fragment by agarose gel electrophoresis. Overexpressed plants were subjected to low-temperature treatment, with WT plants serving as controls.

### Determination of anthocyanin contents

Anthocyanin content was determined following the extraction and detection method described by Zhang *et al*. [2]. In brief, the mortar was precooled with liquid nitrogen, and leaves of *B. semperflorens* stored at −80°C were ground into fine powder under liquid nitrogen immersion. Then, 0.3 g of the powder was transferred to a 2 ml centrifuge tube. Anthocyanins were extracted from the leaf powder by incubation with 1% hydrochloric acid–methanol at 4°C for 12 h. After centrifugation at 12 000 rpm and 4°C for 5 min, the supernatant was collected, and its absorbance was measured at 530 and 657 nm using a spectrophotometer. The relative anthocyanin content in the sample was calculated using the formula: (A_530_ − 0.25 × A_657_).

### Yeast one-hybrid assay

The BsDFR-pHIS2 and BsTT2-pGADT7 constructs were transformed into Y187 yeast strains and cultured on SD/Trp plates. Normal yeast growth confirmed that the plasmid expression products were not toxic to the yeast strains. Colonies containing the BsDFRpro-pHIS2 plasmid were resuspended to an OD600 of 1 and serially diluted 10-fold, 100-fold, and 1000-fold. Diluted samples were spotted onto SD/−Leu/−Trp plates containing 0, 10, 20, 30, 40, 50, 60, 70, 80, 90, and 100 mM of 3-AT to identify the concentration sufficient to inhibit BsDFR promoter activity. pAD-BsTT2 and pHIS2-BsDFRpro were co-transfected into Y187 yeast strains, plated on SD/−Leu/−Trp plates, and resuspended to an OD_600_ of 1. These samples were serially diluted (10-fold, 100-fold, and 1000-fold) and placed on SD/−His/−Leu/−Trp/70 mM 3-AT plates. Results were observed after 72 h of incubation.

### Dual-luciferase assay

The constructs were transformed into *Agrobacterium tumefaciens* GV3101(pSoup) and cultured at 28°C and 120 rpm until OD_600_ reached 1.0–2.0. The bacterial cultures were centrifuged at 5000 rpm for 10 min, resuspended in a solution containing 1 mM MES and 1 mM MgCl_2_, centrifuged again, and adjusted to an OD_600_ of 0.5. The samples were then infiltrated and incubated for 48–72 h. After sampling, the YAsian Dual Luciferase Reporter Gene Assay Kit was used to process the samples, and the values were measured using a microplate reader. Leaf images were captured with a fully automated chemiluminescence image analysis system.

### Transient genetic transformation in apple peels

*BsDFR*, *BsTT2*, and *BsAlfin2* were cloned into the vector pGreenII 62-sk, resulting in the constructs pGreenII 62-sk-BsDFR, pGreenII 62-sk-BsTT2, and pGreenII 62-sk-BsAlfin2. The construct was transformed into *A. tumefaciens* strain GV3101. Positive agrobacterial colonies were cultured in a shaker at 28°C for 24–36 h, with the empty pGreenII 62-sk vector serving as the control. The suspensions of pGreenII 62-sk (empty vector), pGreenII 62-sk-BsDFR alone, pGreenII 62-sk-BsTT2 alone, pGreenII 62-sk-BsAlfin2, and 62-SK-BsTT2 in combination with 62-SK-BsAflin2 were infiltrated into apple peels. The infiltration sites were marked, and the infiltrated apples were kept in darkness for 1 day, then transferred to light conditions for 2–3 days before phenotypic observation.

### Yeast-two-hybrid screening

The construct pGBKT7-BsTT2 was transformed into Y2HGold yeast strains and cultured on SD/Trp plates. Normal yeast growth confirmed successful transformation. The transformed yeast was then plated on SD/−Trp/−Leu/−His/−Ade plates containing 0, 10, 20, 30, 40, 50, 60, 70, and 80 mM 3-AT. Self-activation inhibition was observed after 5 days of culture. The yeast cDNA library was constructed by Shanghai Oe Biotech Co., Ltd, using RNA extracted from leaves of *B. semperflorens* as the template and the pGADT7-DEST vector as the library vector framework, and the total clone number of the cDNA library was ~1.2 × 10^7^, the titer was 6.0 × 10^7^ cfu ml^–1^, the average insert size was >1 kb, and the recombination rate was 96%–100%. The library plasmid and pGBKT7-BsTT2 were co-transformed into Y2HGold yeast strains and plated on SD/−Trp/−Leu/−His/−Ade/80 mM 3-AT/X-α-Gal plates. After 5 days, single colonies were selected for PCR, and the products were sent to Sangon Biotech (Shanghai) Co., Ltd, for sequencing. Genes identified from the sequencing results were cloned into pGADT7 vectors and co-transformed with pGBKT7-BsTT2 into Y2HGold yeast strains. The transformants were then cultured on SD/−Trp/−Leu/−His/−Ade/80 mM 3-AT/X-α-Gal plates for complementary verification, following the same protocol as described above.

### Bimolecular fluorescence complementation

The constructs BsTT2-pCAMBIA1300-35S-nYFP, BsALFIN2-pCAMBIA1300-35S-cYFP, Empty-pCAMBIA1300-35S-nYFP, and Empty-pCAMBIA1300-35S-cYFP were transformed into *A. tumefaciens* GV3101. The lower epidermis of tobacco leaves was observed under a confocal microscope 48–72 h postinjection.

### Statistical analysis

Data are presented as the means ± standard errors (SEs). Differences in outcomes between the treatments were evaluated and visualized by Prism 9 for macOS software (version 9.1.1). Independent-samples Student’s *t*-tests and one-way analysis of variance (ANOVA) were performed to evaluate statistical significance.

## Supplementary Material

Web_Material_uhag319

## Data Availability

The data that supports the findings of this study are available in the supplementary material of this article.
