# Supplementary material for: From genome to gene module: decoding the BsAlfin2-BsTT2 regulatory network controlling low temperature-induced anthocyanin biosynthesis in Begonia semperflorens
Source: Hortic Res. 2026 Aug 6;13(10):uhag319. doi: 10.1093/hr/uhag319 (PMC13634682; doi:10.1093/hr/uhag319)
Supplement: Supplementary file 1 [file web_material_uhag319.docx]

**SUPPORTING INFORMATION**

Figure S1 Leaf color changes of *Begonia semperflorens* under low-temperature treatment. Low-temperature treatment was performed at 15 °C/5 °C (day/night).

Figure S2 Changes of anthocyanin content in leaves of *Begonia semperflorens* under low-temperature treatment. Low-temperature treatment was performed at 15 °C/5 °C (day/night).

Figure S3 23-kmer frequency distribution curve. The x-axis represents the 23-kmer depth (coverage), and the y-axis represents the frequency at each depth.

Figure S4 Comparison of the number of homologous genes between species.

Figure S5 Predicted cis-acting elements in the *BsMYB* promoters in *Begonia semperflorens* genome.

Figure S6 Phylogenetic relationships of the BsMYB gene family members from the genomes of *B. masoniana, B. semperflorens, and Arabidopsis thaliana*.

Figure S7 Chromosomal locations and syntenic relationship of BsMYB proteins.

Figure S8 Collinearity analysis of BsMYB genes between *B. semperflorens* and *A. thaliana* or other *Begonia* species.

Figure S9 The yeast one-hybrid assay revealing the interaction between BsTT2 and *BsCHS*, *BsCHI*, *BsF3H*, *BsANS*, *Bs3GT*, *BsUFGT* promoter.

Figure S10 Images of the dual-LUC assays. The bars on the right represent the intensity of luciferase signal. Red represents higher signal intensity and blue represents lower signal intensity.

Figure S11 Function verify of *BsDFR* in apple. (A) *BsDFR* instantaneous transformation in apple fruit. (B) The relative anthocyanin content in the p35S-Empty and p35S-BsDFR. (C) The qPCR analysis of the anthocyanin biosynthesis genes expression levels in the p35S-Empty and p35S-BsDFR. Three biological replicates were performed. The error bars represent the SD. Asterisk above the bars indicate statistically significant differences (Student’s t-test, ***P* < 0.01, **P* < 0.05).

Figure S12 Phylogenetic comparison of the 50 most similar genes with g14039.t1.

Figure S13 The expression levels of *BsTT2* and *BsAlfin2* in transient overexpression of apple.

Figure S14 Western blot analysis of BsAlfin2 protein levels.

Figure S15 Quantification of relative fluorescence intensity of BsAlfin2 subcellular localization under different treatments.

Table S1 Sequencing information of *B. semperflorens*

| **Lane** | **Raw Base (Gb)** | **Clean Base (Gb)** |
| --- | --- | --- |
| E250048930_L01_CWHPE25050393-69 | 1.29 | 1.27 |
| E250048930_L01_CWHPE25050393-66 | 0.96 | 0.94 |
| E250048930_L01_CWHPE25050393-68 | 0.89 | 0.87 |
| E250048930_L01_CWHPE25050393-72 | 0.95 | 0.94 |
| E250048930_L01_CWHPE25050393-67 | 0.88 | 0.87 |
| E250048930_L01_CWHPE25050393-71 | 0.87 | 0.86 |
| E250048930_L01_CWHPE25050393-70 | 0.78 | 0.76 |
| E250048930_L01_CWHPE25050393-65 | 1.23 | 1.21 |
| E250055907_L01_CWHPE25050393-67 | 2.23 | 2.2 |
| E250055907_L01_CWHPE25050393-68 | 2.22 | 2.19 |
| E250055907_L01_CWHPE25050393-66 | 2.44 | 2.41 |
| E250055907_L01_CWHPE25050393-70 | 2.21 | 2.18 |
| E250055907_L01_CWHPE25050393-69 | 3.40 | 3.35 |
| E250055907_L01_CWHPE25050393-71 | 2.30 | 2.27 |
| E250055907_L01_CWHPE25050393-65 | 3.10 | 3.06 |
| E250055907_L01_CWHPE25050393-72 | 2.51 | 2.48 |
| Total | 28.23 | 27.87 |

Table S2 Pseudomolecule length statistics after Hi-C assisted assembly

| **Chromosome ID** | **Number of contig in chromosome** | **Size (bp)** |
| --- | --- | --- |
| Chr01 | 4 | 24867405 |
| Chr02 | 4 | 24139334 |
| Chr03 | 6 | 22036360 |
| Chr04 | 3 | 19352955 |
| Chr05 | 4 | 18952421 |
| Chr06 | 3 | 15999887 |
| Chr07 | 4 | 14432831 |
| Chr08 | 3 | 12424953 |
| Chr09 | 4 | 12304461 |
| Chr10 | 1 | 12082662 |
| Chr11 | 2 | 11698843 |
| Chr12 | 2 | 11277305 |
| Chr13 | 1 | 11035314 |
| Chr14 | 3 | 10686272 |
| Chr15 | 1 | 9964000 |
| Chr16 | 7 | 9806599 |
| Chr17 | 8 | 8363599 |

Table S3 Statistics of chromosome-level assembly

|  | **Contig** | | **Scaffold** | |
| --- | --- | --- | --- | --- |
|  | **Size (bp)** | **number** | **Size (bp)** | **number** |
| N10 | 19,747,917 | 2 | 24,139,334 | 2 |
| N20 | 16,962,022 | 3 | 22,036,360 | 3 |
| N30 | 12,082,662 | 5 | 19,352,955 | 4 |
| N40 | 11,035,314 | 7 | 18,952,421 | 5 |
| N50 | 8,963,449 | 10 | 14,432,831 | 7 |
| N60 | 7,399,859 | 13 | 12,304,461 | 9 |
| N70 | 6,068,920 | 17 | 11,698,843 | 11 |
| N80 | 3,801,915 | 22 | 11,035,314 | 13 |
| N90 | 2,313,356 | 30 | 9,806,599 | 16 |
| Max Length | 24,028,444 |  | 24,867,405 |  |
| Total Number |  | 80 |  | 29 |
| Total Length | 259,681,412 |  | 259,704,050 |  |
| GC Ratio | 0.38 |  | 0.38 |  |

Table S4 BUSCO assessment of genome assembly of *B. semperflorens*

| **Type** | **Contig level** | | **Chromosome level** | |
| --- | --- | --- | --- | --- |
|  | **Number** | **Percentage (%)** | **Number** | **Percentage (%)** |
| Complete BUSCOs (C) | 1,968 | 97.14 | 1,968 | 97.14 |
| Complete and single-copy BUSCOs (S) | 1,642 | 81.05 | 1,643 | 81.1 |
| Complete and duplicated BUSCOs (D) | 326 | 16.09 | 325 | 16.04 |
| Fragmented BUSCOs (F) | 26 | 1.28 | 26 | 1.28 |
| Missing BUSCOs (M) | 32 | 1.58 | 32 | 1.58 |
| Total BUSCO groups searched | 2,026 |  | 2,026 |  |

Table S5 Functional annotation of *B. semperflorens* genome

| **Database** | **Annotated number** | **Percentage (%)** |
| --- | --- | --- |
| Total | 23,749 | 100.00 |
| Nr | 23,287 | 98.05 |
| Swiss-Prot | 19,405 | 81.71 |
| KEGG | 17,821 | 75.04 |
| KOG | 18,132 | 76.35 |
| TrEMBL | 23,274 | 98.00 |
| Interpro | 22,522 | 94.83 |
| GO | 14,536 | 61.21 |
| Overall | 23,340 | 98.28 |

Table S6 Statistics of repetitive sequences in *B. semperflorens* genome

| **Type** | **Repeat Length (bp)** | **Percentage in genome (%)** |
| --- | --- | --- |
| Trf | 12,495,069.00 | 4.81 |
| Repeatmasker | 29,771,231.00 | 11.46 |
| Proteinmask | 29,493,215.00 | 11.36 |
| De novo | 110,393,169.00 | 42.51 |
| Total | 116,722,590.00 | 44.94 |

Table S7 Classification of repetitive sequences in *B. semperflorens* genome

| **Type** | | | **Length (bp)** | **Percentage in genome (%)** |
| --- | --- | --- | --- | --- |
| Retro | LTR | Copia | 24414049 | 9.40 |
|  |  | Gypsy | 66440706 | 25.58 |
|  |  | Other | 12647350 | 4.87 |
|  | Non-LTR | SINE | 19288 | 0.01 |
|  |  | LINE | 3092016 | 1.19 |
|  | Other | | 0 | 0.00 |
| DNA | EnSpm | | 199269 | 0.08 |
|  | Harbinger | | 202743 | 0.08 |
|  | hAT | | 9193417 | 3.54 |
|  | Helitron | | 805100 | 0.31 |
|  | Mariner | | 202 | 0.00 |
|  | MuDR | | 1141150 | 0.44 |
|  | P | | 10374 | 0.00 |
|  | Other | | 839218 | 0.32 |
| Other | | | 602081 | 0.23 |
| Unknown | | | 5307089 | 2.04 |
| Total | | | 113984382 | 43.89 |

Table S8 Gene family clustering results

| **Species** | **Number of genes** | **Number of genes in OTGs** | **Number of unassigned genes** | **Number of genes in species-specific OTGs** |
| --- | --- | --- | --- | --- |
| *B. darthvaderiana* | 23,245 | 21,364 | 1,881 | 607 |
| *B. loranthoides* | 20,824 | 19,958 | 866 | 1,115 |
| *B. masoniana* | 22,812 | 21,256 | 1,556 | 575 |
| *B. peltatifolia* | 20,497 | 19,682 | 815 | 122 |
| *B. semperflorens* | 23,749 | 22,517 | 1,232 | 570 |
| *C. sativus* | 19,939 | 18,121 | 1,818 | 913 |

Table S9 Physicochemical properties of BsMYB proteins in *B. semperflorens*

| Gene ID | Sequence ID | Number of amino acid | Molecular weight | Theoretical pI | Instability index | Aliphatic index | Grand average of hydropathicity |
| --- | --- | --- | --- | --- | --- | --- | --- |
| BsMYB1 | Bego01G02170.1 | 202 | 22870.57 | 6.06 | 53.01 | 69.55 | -0.726 |
| BsMYB2 | Bego01G02330.1 | 212 | 24196.98 | 6.32 | 57.2 | 68.96 | -0.778 |
| BsMYB3 | Bego01G04620.1 | 220 | 25098.41 | 9.54 | 36.91 | 83.32 | -0.746 |
| BsMYB4 | Bego01G05150.1 | 315 | 36165.17 | 6.23 | 48.37 | 68.38 | -0.743 |
| BsMYB5 | Bego01G06300.1 | 405 | 44507.17 | 6.15 | 53.86 | 65.09 | -0.577 |
| BsMYB6 | Bego01G09120.1 | 753 | 84314.45 | 8.33 | 47.51 | 79.4 | -0.354 |
| BsMYB7 | Bego01G09680.1 | 318 | 36010.41 | 7.66 | 58.87 | 69.31 | -0.672 |
| BsMYB8 | Bego01G09860.1 | 310 | 34889.13 | 7.08 | 57.41 | 68.32 | -0.727 |
| BsMYB9 | Bego01G10590.1 | 276 | 30876.15 | 8.76 | 49.83 | 78.12 | -0.591 |
| BsMYB10 | Bego01G10720.1 | 387 | 43416.97 | 6.18 | 54.44 | 73.82 | -0.568 |
| BsMYB11 | Bego01G11180.1 | 274 | 30290.63 | 9.81 | 43.47 | 69.45 | -0.459 |
| BsMYB12 | Bego01G13810.1 | 265 | 30656.48 | 8.55 | 42.27 | 65.81 | -0.87 |
| BsMYB13 | Bego01G15020.1 | 613 | 68152.08 | 5.45 | 52.47 | 81.24 | -0.443 |
| BsMYB14 | Bego01G16970.1 | 344 | 37933.1 | 5.93 | 60.47 | 60.09 | -0.785 |
| BsMYB15 | Bego01G17320.1 | 173 | 19641.51 | 9.51 | 37.94 | 83.47 | -0.68 |
| BsMYB16 | Bego01G18980.1 | 339 | 37507.83 | 6.28 | 59.2 | 66.17 | -0.714 |
| BsMYB17 | Bego01G19870.1 | 102 | 11942.89 | 9.62 | 60.98 | 72.65 | -0.695 |
| BsMYB18 | Bego01G26740.1 | 298 | 33815.98 | 8.39 | 53.64 | 65.44 | -0.791 |
| BsMYB19 | Bego01G27040.1 | 275 | 31245.12 | 8.09 | 62.6 | 62.07 | -0.891 |
| BsMYB20 | Bego01G27050.1 | 263 | 29923.58 | 5.25 | 48.54 | 66.39 | -0.669 |
| BsMYB21 | Bego01G27060.1 | 231 | 26274.77 | 7.65 | 51.74 | 74.81 | -0.682 |
| BsMYB22 | Bego01G29220.1 | 290 | 33143.07 | 8.76 | 51.32 | 61.83 | -0.827 |
| BsMYB23 | Bego01G31370.1 | 538 | 60927.21 | 6.08 | 54.93 | 71.99 | -0.697 |
| BsMYB24 | Bego02G00230.1 | 248 | 28596.78 | 9.13 | 50.41 | 73.99 | -0.65 |
| BsMYB25 | Bego02G02740.1 | 339 | 38239.01 | 4.91 | 60.74 | 63.54 | -0.91 |
| BsMYB26 | Bego02G04120.1 | 252 | 28015.56 | 9.3 | 73.74 | 68.97 | -0.774 |
| BsMYB27 | Bego02G04390.1 | 271 | 30248.25 | 9.19 | 51.76 | 64.83 | -0.701 |
| BsMYB28 | Bego02G04440.1 | 281 | 31235.38 | 4.81 | 48.34 | 65.98 | -0.765 |
| BsMYB29 | Bego02G04710.1 | 385 | 43260.51 | 7.75 | 56.51 | 71.4 | -0.728 |
| BsMYB30 | Bego02G05640.1 | 287 | 32494.42 | 7.61 | 51.59 | 64.91 | -0.753 |
| BsMYB31 | Bego02G05750.1 | 294 | 32593.36 | 5.25 | 56.94 | 69.66 | -0.598 |
| BsMYB32 | Bego02G05780.1 | 261 | 28604.07 | 9.92 | 49.69 | 72.53 | -0.546 |
| BsMYB33 | Bego02G07650.1 | 356 | 40079.59 | 4.96 | 63.1 | 71.74 | -0.667 |
| BsMYB34 | Bego02G08950.1 | 96 | 11206.93 | 9.08 | 65.3 | 81.25 | -0.578 |
| BsMYB35 | Bego02G09050.1 | 256 | 28894.87 | 8.71 | 50.5 | 73.87 | -0.621 |
| BsMYB36 | Bego02G09060.1 | 196 | 23217.34 | 9.55 | 75.33 | 66.22 | -1.048 |
| BsMYB37 | Bego02G09550.1 | 510 | 56079.78 | 6.65 | 52.23 | 65.12 | -0.409 |
| BsMYB38 | Bego03G00550.1 | 310 | 34963.53 | 8.08 | 53.46 | 70.16 | -0.655 |
| BsMYB39 | Bego03G02100.1 | 407 | 45371.98 | 5.57 | 63.82 | 76.22 | -0.612 |
| BsMYB40 | Bego03G02570.1 | 224 | 25582.69 | 5.06 | 50.72 | 80.13 | -0.575 |
| BsMYB41 | Bego03G04290.1 | 238 | 26842.85 | 5.09 | 54.68 | 66.43 | -0.497 |
| BsMYB42 | Bego03G05510.1 | 343 | 37593.98 | 6.68 | 64.69 | 64.26 | -0.729 |
| BsMYB43 | Bego03G08880.1 | 239 | 27540.01 | 9.23 | 59.38 | 67.24 | -0.894 |
| BsMYB44 | Bego03G09190.1 | 173 | 19686.46 | 9.51 | 35.09 | 77.86 | -0.74 |
| BsMYB45 | Bego03G09480.1 | 530 | 58454.51 | 4.86 | 41.51 | 66.45 | -0.618 |
| BsMYB46 | Bego03G09550.1 | 198 | 22432.1 | 6.24 | 47.36 | 70.35 | -0.681 |
| BsMYB47 | Bego03G10140.1 | 293 | 34078.22 | 8.85 | 56.76 | 69.86 | -0.764 |
| BsMYB48 | Bego03G11050.1 | 245 | 27590.33 | 8.9 | 54.82 | 76.53 | -0.627 |
| BsMYB49 | Bego03G11420.1 | 413 | 45297.78 | 5.92 | 58.96 | 71.65 | -0.519 |
| BsMYB50 | Bego03G12050.1 | 327 | 35782.65 | 9.17 | 59.77 | 68.01 | -0.593 |
| BsMYB51 | Bego03G12310.1 | 89 | 10816.29 | 7.79 | 88.42 | 71.24 | -0.918 |
| BsMYB52 | Bego03G13110.1 | 446 | 49327.22 | 6.55 | 54.48 | 66.91 | -0.681 |
| BsMYB53 | Bego03G14330.1 | 325 | 36528.74 | 5.94 | 45.05 | 63.66 | -0.636 |
| BsMYB54 | Bego03G14680.1 | 640 | 70980.81 | 5.98 | 39.87 | 73.56 | -0.494 |
| BsMYB55 | Bego03G15080.1 | 348 | 39023.5 | 6.61 | 59.98 | 68.16 | -0.712 |
| BsMYB56 | Bego03G16080.1 | 277 | 30229.79 | 9.2 | 54.65 | 67.87 | -0.565 |
| BsMYB57 | Bego03G18430.1 | 334 | 37630 | 5.94 | 47.82 | 69.22 | -0.628 |
| BsMYB58 | Bego03G19260.1 | 106 | 12207.43 | 10.21 | 59.21 | 90.19 | -0.375 |
| BsMYB59 | Bego03G19390.1 | 273 | 30257.94 | 9.05 | 32.62 | 72.64 | -0.76 |
| BsMYB60 | Bego03G20400.1 | 318 | 35953.9 | 8.24 | 57.35 | 54.62 | -0.886 |
| BsMYB61 | Bego03G21240.1 | 211 | 24223.2 | 8.68 | 71.65 | 59.15 | -0.992 |
| BsMYB62 | Bego03G21710.1 | 287 | 31997.68 | 8.57 | 56.87 | 67.6 | -0.671 |
| BsMYB63 | Bego03G22130.1 | 614 | 69025.86 | 5.92 | 43.79 | 83.7 | -0.49 |
| BsMYB64 | Bego03G23390.1 | 430 | 47756.69 | 5.68 | 58.04 | 73.47 | -0.524 |
| BsMYB65 | Bego03G25480.1 | 314 | 35262.55 | 6.6 | 46.06 | 73.98 | -0.567 |
| BsMYB66 | Bego03G28550.1 | 249 | 28972.53 | 5.88 | 69.17 | 69.68 | -0.629 |
| BsMYB67 | Bego03G30130.1 | 320 | 35625.96 | 6.68 | 62.74 | 67.34 | -0.677 |
| BsMYB68 | Bego04G00190.1 | 257 | 28686.73 | 7.83 | 47.09 | 80.47 | -0.588 |
| BsMYB69 | Bego04G00660.1 | 295 | 33052.25 | 6.19 | 59.53 | 70.47 | -0.66 |
| BsMYB70 | Bego04G01190.1 | 279 | 31457.22 | 5.7 | 55.53 | 63.66 | -0.715 |
| BsMYB71 | Bego04G03270.1 | 74 | 8664.78 | 8.03 | 64.81 | 65.95 | -0.936 |
| BsMYB72 | Bego04G04100.1 | 408 | 45278.44 | 6.89 | 62.79 | 65.1 | -0.655 |
| BsMYB73 | Bego04G07460.1 | 191 | 22268.24 | 8.42 | 56.99 | 59.69 | -0.928 |
| BsMYB74 | Bego04G07620.1 | 88 | 10039.46 | 8.9 | 47.35 | 67.61 | -0.839 |
| BsMYB75 | Bego04G09080.1 | 582 | 64810.98 | 5.64 | 51.8 | 82.22 | -0.489 |
| BsMYB76 | Bego04G10010.1 | 640 | 71675.81 | 8.28 | 56.12 | 60.3 | -0.884 |
| BsMYB77 | Bego04G10700.1 | 326 | 37123.58 | 5.54 | 53.49 | 73.34 | -0.635 |
| BsMYB78 | Bego04G10710.1 | 240 | 27297.65 | 8.33 | 63.56 | 62.63 | -0.765 |
| BsMYB79 | Bego05G01160.1 | 307 | 34495.96 | 6.32 | 55.49 | 73.39 | -0.532 |
| BsMYB80 | Bego05G02810.1 | 320 | 36553.07 | 6.32 | 42.41 | 76.5 | -0.703 |
| BsMYB81 | Bego05G03430.1 | 365 | 40357.35 | 6.22 | 54.01 | 75.62 | -0.57 |
| BsMYB82 | Bego05G04360.1 | 264 | 30055.74 | 9.3 | 46.23 | 65.42 | -0.872 |
| BsMYB83 | Bego05G05050.1 | 287 | 32629.41 | 8.74 | 50.73 | 55.05 | -0.859 |
| BsMYB84 | Bego05G05310.1 | 478 | 54000.52 | 7.62 | 46.76 | 51.42 | -1.04 |
| BsMYB85 | Bego05G07370.1 | 270 | 30763.67 | 7.62 | 46.78 | 66.85 | -0.722 |
| BsMYB86 | Bego05G07380.1 | 188 | 20821.31 | 4.81 | 64.16 | 71.12 | -0.581 |
| BsMYB87 | Bego05G07680.1 | 279 | 32597.7 | 9.54 | 59.04 | 52.47 | -1.006 |
| BsMYB88 | Bego05G07820.1 | 260 | 29626.69 | 6.16 | 50.74 | 76.92 | -0.646 |
| BsMYB89 | Bego05G08230.1 | 269 | 30140.8 | 7.03 | 40.68 | 70.67 | -0.774 |
| BsMYB90 | Bego05G09260.1 | 199 | 23070.87 | 6.24 | 57.08 | 74.42 | -0.891 |
| BsMYB91 | Bego05G12670.1 | 250 | 28888.48 | 7.65 | 62.14 | 52.68 | -0.888 |
| BsMYB92 | Bego05G13710.1 | 224 | 25900.95 | 6.25 | 53.28 | 68.3 | -0.884 |
| BsMYB93 | Bego05G14690.1 | 315 | 35267.39 | 7.21 | 55.35 | 74.03 | -0.857 |
| BsMYB94 | Bego05G17070.1 | 366 | 41577.5 | 8.59 | 51.15 | 63.42 | -0.881 |
| BsMYB95 | Bego05G18370.1 | 403 | 44604.52 | 5.52 | 62.86 | 63.62 | -0.814 |
| BsMYB96 | Bego05G19880.1 | 353 | 38832.07 | 6.56 | 58.28 | 66.91 | -0.728 |
| BsMYB97 | Bego05G19990.1 | 268 | 30155.95 | 8.5 | 53.65 | 67.39 | -0.756 |
| BsMYB98 | Bego05G20790.1 | 190 | 21973.67 | 8.72 | 50.08 | 69.37 | -0.851 |
| BsMYB99 | Bego05G21840.1 | 545 | 61652.58 | 9.16 | 46.54 | 72.51 | -0.674 |
| BsMYB100 | Bego05G22080.1 | 381 | 42421.73 | 5.96 | 48.87 | 49.24 | -0.738 |
| BsMYB101 | Bego06G00190.1 | 309 | 35200.63 | 5.44 | 59.68 | 74.47 | -0.552 |
| BsMYB102 | Bego06G00360.1 | 253 | 29073.9 | 5.31 | 61.62 | 77.47 | -0.68 |
| BsMYB103 | Bego06G00540.1 | 274 | 31179.08 | 9.59 | 56.83 | 57.63 | -0.803 |
| BsMYB104 | Bego06G01350.1 | 304 | 33925.79 | 6 | 59.48 | 71.91 | -0.837 |
| BsMYB105 | Bego06G03050.1 | 213 | 24321.12 | 7.02 | 51.73 | 71.36 | -0.845 |
| BsMYB106 | Bego06G03320.1 | 309 | 34798.13 | 6.26 | 57.11 | 68.74 | -0.588 |
| BsMYB107 | Bego06G03350.1 | 241 | 26771.78 | 4.97 | 43.91 | 70.75 | -0.477 |
| BsMYB108 | Bego06G03810.1 | 308 | 34657.22 | 5.84 | 52.78 | 73.21 | -0.642 |
| BsMYB109 | Bego06G06740.1 | 252 | 28797.25 | 6.46 | 52.98 | 66.94 | -0.732 |
| BsMYB110 | Bego06G07980.1 | 293 | 32178.54 | 6.41 | 51.03 | 80.58 | -0.462 |
| BsMYB111 | Bego06G08180.1 | 191 | 21905.69 | 6.16 | 58.8 | 79.11 | -0.543 |
| BsMYB112 | Bego06G08460.1 | 299 | 33708.43 | 5.79 | 56.05 | 65.59 | -0.678 |
| BsMYB113 | Bego06G09250.1 | 368 | 40787.21 | 5.18 | 53.37 | 74.73 | -0.592 |
| BsMYB114 | Bego06G11430.1 | 320 | 36181.77 | 6.72 | 68.65 | 69.72 | -0.595 |
| BsMYB115 | Bego06G12060.1 | 275 | 31085.09 | 6.4 | 52.41 | 69.85 | -0.689 |
| BsMYB116 | Bego06G12860.1 | 243 | 27182.69 | 9.59 | 50.03 | 59.84 | -0.796 |
| BsMYB117 | Bego06G15930.1 | 273 | 30833.66 | 6.33 | 59.19 | 72.2 | -0.657 |
| BsMYB118 | Bego06G15940.1 | 281 | 31649.47 | 6.9 | 47.57 | 77.79 | -0.672 |
| BsMYB119 | Bego06G15950.1 | 223 | 25482.29 | 5.69 | 57.15 | 62.91 | -0.895 |
| BsMYB120 | Bego06G16580.1 | 277 | 32033.79 | 5.86 | 59.68 | 71.81 | -0.802 |
| BsMYB121 | Bego06G17610.1 | 351 | 39846.38 | 6.04 | 56.58 | 56.41 | -0.68 |
| BsMYB122 | Bego07G00180.1 | 253 | 28834.44 | 5.45 | 54.19 | 68.66 | -0.71 |
| BsMYB123 | Bego07G01900.1 | 231 | 26932.22 | 6.47 | 70.11 | 56.58 | -0.95 |
| BsMYB124 | Bego07G03990.1 | 400 | 44640.26 | 5.34 | 61.47 | 65.57 | -0.911 |
| BsMYB125 | Bego07G04300.1 | 1013 | 114117.49 | 8.82 | 50.86 | 71.9 | -0.69 |
| BsMYB126 | Bego07G04810.1 | 190 | 21772.47 | 6.44 | 56.86 | 78.05 | -0.726 |
| BsMYB127 | Bego07G05330.1 | 250 | 28410.17 | 9.69 | 46.45 | 63.92 | -0.838 |
| BsMYB128 | Bego07G06230.1 | 313 | 34214.8 | 7.09 | 38.63 | 77.28 | -0.392 |
| BsMYB129 | Bego07G06580.1 | 224 | 25080.11 | 8.14 | 55.53 | 57.9 | -0.704 |
| BsMYB130 | Bego07G07960.1 | 394 | 45204.68 | 7.19 | 64.63 | 70.81 | -0.726 |
| BsMYB131 | Bego07G08900.1 | 256 | 28957.47 | 8.85 | 46.9 | 62.85 | -0.871 |
| BsMYB132 | Bego07G08990.1 | 219 | 24703.67 | 7.78 | 57.95 | 64.16 | -1.023 |
| BsMYB133 | Bego07G09230.1 | 622 | 70499.46 | 8.86 | 64.39 | 65.51 | -0.805 |
| BsMYB134 | Bego07G10130.1 | 288 | 32830 | 6.18 | 47.26 | 79.27 | -0.581 |
| BsMYB135 | Bego07G12930.1 | 188 | 22026.59 | 7.73 | 67.19 | 62.34 | -1.111 |
| BsMYB136 | Bego07G12950.1 | 260 | 29454.64 | 8.53 | 56.36 | 80.96 | -0.534 |
| BsMYB137 | Bego07G13100.1 | 296 | 33138.46 | 6.4 | 63.65 | 79.73 | -0.504 |
| BsMYB138 | Bego07G13950.1 | 291 | 32982.96 | 5.19 | 59.76 | 61.37 | -0.68 |
| BsMYB139 | Bego08G00160.1 | 661 | 72833.51 | 5.75 | 42.19 | 75.49 | -0.507 |
| BsMYB140 | Bego08G03190.1 | 300 | 33949.79 | 6.26 | 50.66 | 70.23 | -0.659 |
| BsMYB141 | Bego08G03950.1 | 296 | 33733.81 | 5.37 | 65.25 | 58.95 | -0.662 |
| BsMYB142 | Bego08G04050.1 | 273 | 30569.61 | 9.15 | 36.77 | 74.4 | -0.725 |
| BsMYB143 | Bego08G06240.1 | 258 | 28199.62 | 6.76 | 50.42 | 81.32 | -0.588 |
| BsMYB144 | Bego08G10220.1 | 320 | 35604.26 | 6.31 | 46.94 | 68 | -0.62 |
| BsMYB145 | Bego08G12510.1 | 263 | 29732.16 | 8.11 | 60.48 | 59.35 | -0.744 |
| BsMYB146 | Bego08G12970.1 | 247 | 28454.14 | 6.04 | 62.13 | 60.4 | -0.691 |
| BsMYB147 | Bego08G13760.1 | 243 | 28263.79 | 5.64 | 64.65 | 70.66 | -0.869 |
| BsMYB148 | Bego09G00280.1 | 301 | 34737.01 | 9.82 | 52.97 | 53.89 | -0.895 |
| BsMYB149 | Bego09G01290.1 | 249 | 28749.35 | 6.13 | 57.15 | 63.9 | -0.798 |
| BsMYB150 | Bego09G01300.1 | 259 | 29495.98 | 5.59 | 71.18 | 64.4 | -0.893 |
| BsMYB151 | Bego09G01810.1 | 277 | 30719.38 | 7.08 | 61.89 | 69.75 | -0.614 |
| BsMYB152 | Bego09G06660.1 | 197 | 21538.06 | 8.43 | 46.1 | 67.87 | -0.551 |
| BsMYB153 | Bego09G07280.1 | 571 | 64423.81 | 6.13 | 40.38 | 66.94 | -0.65 |
| BsMYB154 | Bego09G08150.1 | 360 | 38991.44 | 5.9 | 44.49 | 70.14 | -0.533 |
| BsMYB155 | Bego09G08250.1 | 277 | 31733.17 | 6.67 | 57.13 | 57.4 | -0.899 |
| BsMYB156 | Bego09G08400.1 | 298 | 34518.98 | 5.33 | 59.47 | 61.21 | -0.887 |
| BsMYB157 | Bego09G10350.1 | 316 | 36198.91 | 9.47 | 47.2 | 67.59 | -0.886 |
| BsMYB158 | Bego09G13790.1 | 273 | 30819.66 | 7.63 | 51 | 63.96 | -0.762 |
| BsMYB159 | Bego09G13820.1 | 326 | 36193.59 | 9.69 | 62.73 | 67.09 | -0.648 |
| BsMYB160 | Bego09G14000.1 | 387 | 42651.75 | 6.86 | 50.87 | 72.61 | -0.653 |
| BsMYB161 | Bego09G14560.1 | 234 | 27151.65 | 6.46 | 52.63 | 64.19 | -0.785 |
| BsMYB162 | Bego09G14980.1 | 343 | 39136.82 | 5.66 | 42.28 | 78.19 | -0.673 |
| BsMYB163 | Bego09G15410.1 | 445 | 49148.5 | 9.67 | 45.88 | 61.64 | -0.681 |
| BsMYB164 | Bego10G03940.1 | 334 | 37495.53 | 6.08 | 47.47 | 74.25 | -0.47 |
| BsMYB165 | Bego10G05430.1 | 303 | 34259.58 | 6.47 | 55.52 | 67 | -0.709 |
| BsMYB166 | Bego10G05690.1 | 304 | 33189.26 | 7.16 | 53.82 | 71.84 | -0.485 |
| BsMYB167 | Bego10G05830.1 | 211 | 24572.7 | 9.35 | 55.58 | 66.02 | -0.825 |
| BsMYB168 | Bego10G06020.1 | 310 | 34772 | 6.26 | 61.55 | 67.94 | -0.563 |
| BsMYB169 | Bego10G06790.1 | 86 | 9722.57 | 4.42 | 54.85 | 64.65 | -0.442 |
| BsMYB170 | Bego10G06840.1 | 456 | 50037.78 | 5.77 | 52.12 | 62.96 | -0.654 |
| BsMYB171 | Bego10G08040.1 | 222 | 25075.59 | 9.46 | 55.46 | 75.23 | -0.725 |
| BsMYB172 | Bego10G11520.1 | 388 | 42838.9 | 5.93 | 41.99 | 62.84 | -0.678 |
| BsMYB173 | Bego10G11810.1 | 409 | 45698.39 | 7.65 | 53.02 | 76.8 | -0.581 |
| BsMYB174 | Bego10G12700.1 | 119 | 13667.43 | 6.62 | 47.91 | 64.79 | -0.894 |
| BsMYB175 | Bego10G13460.1 | 1412 | 155752.54 | 5.86 | 47.19 | 61.66 | -0.791 |
| BsMYB176 | Bego10G14170.1 | 451 | 49528.18 | 5.22 | 54.69 | 67.12 | -0.544 |
| BsMYB177 | Bego11G01510.1 | 278 | 31949.06 | 8.97 | 44.72 | 67.37 | -0.921 |
| BsMYB178 | Bego11G03330.1 | 226 | 25737.06 | 7.08 | 55.98 | 70.31 | -0.719 |
| BsMYB179 | Bego11G06700.1 | 301 | 34297.72 | 5.93 | 49.71 | 72.96 | -0.611 |
| BsMYB180 | Bego11G09210.1 | 238 | 26520.2 | 9.01 | 51.7 | 75.08 | -0.687 |
| BsMYB181 | Bego12G00780.1 | 327 | 37010.7 | 5.93 | 45.09 | 82.02 | -0.554 |
| BsMYB182 | Bego12G00800.1 | 242 | 27950.15 | 6.08 | 60.57 | 59.26 | -0.757 |
| BsMYB183 | Bego12G01070.1 | 408 | 44412.88 | 6.3 | 57.5 | 73.14 | -0.506 |
| BsMYB184 | Bego12G01620.1 | 742 | 82515.44 | 6.05 | 61.94 | 62.48 | -0.889 |
| BsMYB185 | Bego12G02250.1 | 291 | 32326.85 | 8.25 | 48.24 | 73.02 | -0.562 |
| BsMYB186 | Bego12G03570.1 | 304 | 34030.48 | 8.12 | 57.83 | 71.61 | -0.592 |
| BsMYB187 | Bego12G03830.1 | 409 | 45776.25 | 6.98 | 41.46 | 67.92 | -0.59 |
| BsMYB188 | Bego12G04740.1 | 307 | 34671.53 | 5.72 | 48.77 | 65.86 | -0.605 |
| BsMYB189 | Bego12G04920.1 | 302 | 33800.62 | 5.64 | 52.9 | 68.48 | -0.681 |
| BsMYB190 | Bego12G07850.1 | 422 | 47556.72 | 9.03 | 58.23 | 66.59 | -0.662 |
| BsMYB191 | Bego12G07920.1 | 254 | 29656.52 | 5.43 | 59.13 | 69.8 | -0.822 |
| BsMYB192 | Bego12G08840.1 | 311 | 34827.75 | 5.94 | 53.78 | 61.54 | -0.772 |
| BsMYB193 | Bego12G10290.1 | 286 | 32501.23 | 9.42 | 63.85 | 75.38 | -0.765 |
| BsMYB194 | Bego12G11800.1 | 543 | 58263.83 | 5.18 | 51.03 | 77.83 | -0.537 |
| BsMYB195 | Bego13G00440.1 | 261 | 29736.8 | 9.05 | 56.53 | 68.77 | -0.765 |
| BsMYB196 | Bego13G00450.1 | 222 | 25090.68 | 9.45 | 50.21 | 82.25 | -0.552 |
| BsMYB197 | Bego13G01870.1 | 452 | 49050.91 | 8.11 | 52.55 | 60.33 | -0.521 |
| BsMYB198 | Bego13G05350.1 | 200 | 23020.86 | 7.6 | 60.63 | 62.95 | -0.835 |
| BsMYB199 | Bego13G07010.1 | 410 | 45348.56 | 5.57 | 53.14 | 60.2 | -0.921 |
| BsMYB200 | Bego13G08030.1 | 275 | 31451.43 | 5.53 | 48.26 | 72.04 | -0.668 |
| BsMYB201 | Bego13G08990.1 | 354 | 39199.54 | 6.87 | 59.62 | 67.01 | -0.693 |
| BsMYB202 | Bego13G09570.1 | 220 | 25114.91 | 5.22 | 61.09 | 69.95 | -0.673 |
| BsMYB203 | Bego13G09580.1 | 220 | 25069.84 | 5.12 | 60.89 | 71.73 | -0.65 |
| BsMYB204 | Bego13G09850.1 | 116 | 13452.73 | 9.73 | 56.76 | 100 | -0.303 |
| BsMYB205 | Bego14G00670.1 | 947 | 105198.27 | 5.49 | 56.58 | 73.32 | -0.602 |
| BsMYB206 | Bego14G01840.1 | 93 | 10568.69 | 7.78 | 65.52 | 54.52 | -0.957 |
| BsMYB207 | Bego14G02110.1 | 311 | 34262.24 | 7.19 | 53.1 | 70.32 | -0.636 |
| BsMYB208 | Bego14G02440.1 | 252 | 29015.44 | 6.4 | 64 | 65 | -0.923 |
| BsMYB209 | Bego14G02660.1 | 368 | 41334.06 | 9.42 | 51.72 | 59.1 | -0.971 |

Table S10 List of primers used for constructing the vector

| Primer name | Primer sequence (5’-3’) |
| --- | --- |
| BsDFRpro-F | GCCATTTCTGAAGCAGCGTGAGG |
| BsDFRpro-R | GCGAAACGGTCAAAGTCAAGGTC |
| BsDFRpro-SmaI-pHis2-F | ACTATAGGGCGAATTCCCGGGCCTAACTCTGATGGTGGCCTAATG |
| BsDFRpro-SmaI-pHis2-R | CGAACGCGTGAGCTCCCCGGGCGATTGTCGACGTCTGATTAATCA |
| BsDFRpro-SmaI-pGreenII 0800-LUC-F | ATCGAATTCCTGCAGCCCGGGCCTAACTCTGATGGTGGCCTAATG |
| BsDFRpro-SmaI-pGreenII 0800-LUC-R | AGAACTAGTGGATCCCCCGGGCGATTGTCGACGTCTGATTAATCA |
| BsTT2-F | ATGGGGAGGAGGCCGTGCTGCTCC |
| BsTT2-R | TCAAGGCCAGGGTTGAGGATC |
| BsTT2-SmaI-pGADT7-F | GGCCAGTGAATTCCACCCGGGATGGGGAGGAGGCCGTGC |
| BsTT2-SmaI-pGADT7-R | CCGTATCGATGCCCACCCGGGTCAAGGCCAGGGTTGAGGA |
| BsTT2-SmaI-pGBKT7-F | CATGGAGGCCGAATTCCCGGGATGGGGAGGAGGCCGTGC |
| BsTT2-SmaI-pGBKT7-R | GCAGGTCGACGGATCCCCGGGTCAAGGCCAGGGTTGAGGA |
| BsTT2-SmaI-pGreenII 62-SK-F | AGAACTAGTGGATCCCCCGGGATGGGGAGGAGGCCGTGC |
| BsTT2-SmaI-pGreenII 62-SK-R | ATCGAATTCCTGCAGCCCGGGTCAAGGCCAGGGTTGAGGA |
| BsTT2-SmaI-pCanG-F | CTGTACAAGCGGTACCCCGGGATGGGGAGGAGGCCGTGC |
| BsTT2-SmaI-pCanG-R | TCCATCGATTTCGAACCCGGGTCAAGGCCAGGGTTGAGGA |
| BsAlfin2-F | ATGGCTTCCATTTCTTCAAGCCC |
| BsAlfin2-R | CTAAGGCCGGGTCTTCTTTGTCG |
| BsAlfin2-SmaI-pGADT7-F | GGCCAGTGAATTCCACCCGGGATGGCTTCCATTTCTTCAAGCC |
| BsAlfin2-SmaI-pGADT7-R | CCGTATCGATGCCCACCCGGGCTAAGGCCGGGTCTTCTTTGT |
| BsAlfin2-SmaI-pGreenII 62-SK-F | AGAACTAGTGGATCCCCCGGGATGGCTTCCATTTCTTCAAGCC |
| BsAlfin2-SmaI-pGreenII 62-SK-R | ATCGAATTCCTGCAGCCCGGGCTAAGGCCGGGTCTTCTTTGT |
| BsAlfin2-SmaI-pCanG-F | CTGTACAAGCGGTACCCCGGGATGGCTTCCATTTCTTCAAGCCC |
| BsAlfin2-SmaI-pCanG-R | TCCATCGATTTCGAACCCGGGCTAAGGCCGGGTCTTCTTTGTCG |
| pGADT7-F | CTATTCGATGATGAAGATACCCCACCAAACC |
| pGADT7-R | GTGAACTTGCGGGGTTTTTCAGTATCTACGAT |
| pGBKT7-F | GAGGAGCAGAAGCTGATCTCAGAGGAGG |
| pGBKT7-R | AAAACCCCTCAAGACCCGTTTAGAGGCCC |
| pCanG-F | CCAGTCCGCCCTGAGCAAAGACCCCAA |
| pCanG-R | CGTCATGCATTACATGTTAATTATTACATGC |
| BsTT2-pCanG-R2 | TCTCCACTTGCCTTCTCCATGGGCTTCG |
| pGreenII-62-SK-F | GTCATTTCATTTGGAGAGGACAGCCCAAG |
| pGreenII-62-SK-R | CAGCGTACCGAATTGGTACCGGGCC |
| pGreenII 0800-LUC-F | AAAACGACGGCCAGTGAATTGTAATAC |
| pGreenII 0800-LUC-R | CTTTATGTTTTTGGCGTCTTCCATGGAATTC |
| pHis2-F | AATCGATCCGCGGTCTAGAAATTCCTGG |
| pHis2-R | TTTCCCAGTCACGACGTTGTAAAACGACGG |

Table S11 List of primers used for qPCR

| Primer name | Sequence (5'-3') |
| --- | --- |
| qPCR-*BsCHS*-F | GCTCGTGTTCTGGTGGTCTGTTC |
| qPCR-*BsCHS*-R | AGAATTGTTTGGGCAGCGGAGAC |
| qPCR-*BsCHI*-F | TGCTGTGAGAGATCGACTGG |
| qPCR-*BsCHI*-R | GAAAACGCAATCTCGACGGT |
| qPCR-*BsF3H*-F | GTGACCTATTTCTCGTACCCAATCC |
| qPCR-*BsF3H*-R | CCTCCGACAACACCTCCAGTAG |
| qPCR-*BsDFR-*F | TTTCCATGTCGCTACTCCCA |
| qPCR-*BsDFR*-R | ATTCAACGTCGCTCCAATCG |
| qPCR-*BsANS-*F | GCGTGCCCAACTCCATAA |
| qPCR-*BsANS*-R | ACAGCCCAAGAAATCCTC |
| qPCR-*BsUFGT-*F | GCTTGATTGCTCTGACTCCG |
| qPCR-*BsUFGT*-R | AGCTGAGTCACCGAGTTGAA |
| qPCR-*BsTT2-*F | CAACCCTGTTTCTTCACCGATTCC |
| qPCR-*BsTT2-*R | GTGCTCACCTGATTCTCCGTCTC |
| qPCR-*BsMYB298-*F | CCTTGGACGGAAGAAGAACACAG |
| qPCR-*BsMYB298-*R | CATGGCTTGCTACTTGGGTTGG |
| qPCR-*BsMYB326-*F | GTCTGCGATGGATGAACTATCTACG |
| qPCR-*BsMYB326-*R | CAGGCGATGAAGACGAAGAATGAG |
| qPCR-*BsMYB321-*F | AGAGCAGGTCTGAGCAGATGTG |
| qPCR-*BsMYB321-*R | TGTTGCCGAGTAGTTTGTGAAGTC |
| qPCR-*BsMYB343-*F | TGGTTGTTGACATTATCGGAGGAAG |
| qPCR-*BsMYB343-*R | GAGATTGCCATTCGCCATTGTTG |
